# LongShengZhi Capsule Attenuates Alzheimer-Like Pathology in APP/PS1 Double Transgenic Mice by Reducing Neuronal Oxidative Stress and Inflammation

**DOI:** 10.3389/fnagi.2020.582455

**Published:** 2020-11-23

**Authors:** Zequn Yin, Xuerui Wang, Shihong Zheng, Peichang Cao, Yuanli Chen, Maoyun Yu, Chenzhong Liao, Zhongyuan Zhang, Jihong Han, Yajun Duan, Xiaoxiao Yang, Shuang Zhang

**Affiliations:** ^1^Key Laboratory of Metabolism and Regulation for Major Diseases of Anhui Higher Education Institutes, College of Food and Biological Engineering, Hefei University of Technology, Hefei, China; ^2^School of Biological and Pharmaceutical Engineering, West Anhui University, Lu’an, China; ^3^People’s Hospital of Zunhua, Tangshan, China; ^4^College of Life Sciences, Key Laboratory of Medicinal Chemical Biology, Key Laboratory of Bioactive Materials of Ministry of Education, Nankai University, Tianjin, China

**Keywords:** LongShengZhi capsule, Alzheimer’s disease, oxidative stress, amyloid-β, Tau, cognition

## Abstract

Alzheimer’s disease (AD) is the most common form of dementia in the elderly. It may be caused by oxidative stress, inflammation, and cerebrovascular dysfunctions in the brain. LongShengZhi Capsule (LSZ), a traditional Chinese medicine, has been approved by the China Food and Drug Administration for treatment of patients with cardiovascular/cerebrovascular disease. LSZ contains several neuroprotective ingredients, including *Hirudo, Astmgali Radix, Carthami Flos (Honghua), Persicae Semen (Taoren), Acori Tatarinowii Rhizoma (Shichangpu)*, and *Acanthopanax Senticosus (Ciwujia)*. In this study, we aimed to determine the effect of LSZ on the AD process. Double transgenic mice expressing the amyloid-β precursor protein and mutant human presenilin 1 (APP/PS1) to model AD were treated with LSZ for 7 months starting at 2 months of age. LSZ significantly improved the cognition of the mice without adverse effects, indicating its high degree of safety and efficacy after a long-term treatment. LSZ reduced AD biomarker Aβ plaque accumulation by inhibiting β-secretase and γ-secretase gene expression. LSZ also reduced p-Tau expression, cell death, and inflammation in the brain. Consistently, *in vitro*, LSZ ethanol extract enhanced neuronal viability by reducing L-glutamic acid-induced oxidative stress and inflammation in HT-22 cells. LSZ exerted antioxidative effects by enhancing superoxide dismutase and glutathione peroxidase expression, reduced Aβ accumulation by inhibiting β-secretase and γ-secretase mRNA expression, and decreased p-Tau level by inhibiting NF-κB-mediated inflammation. It also demonstrated neuroprotective effects by regulating the Fas cell surface death receptor/B-cell lymphoma 2/p53 pathway. Taken together, our study demonstrates the antioxidative stress, anti-inflammatory, and neuroprotective effects of LSZ in the AD-like pathological process and suggests it could be a potential medicine for AD treatment.

## Introduction

Alzheimer’s disease (AD) is a disease accompanied by behavioral and cognitive impairment, which potently affects the normal social life of the elderly. More than 40 million people worldwide suffer from AD, and many eventually die from serious complications, such as cerebral thrombosis, aspiration pneumonia, and heart failure (Jankowska et al., 2019). Amyloid-β (Aβ) plaques formed by aggregation of Aβ monomer and neurofibrillary tangles (NFTs) formed by hyperphosphorylation of microtubule-associated protein Tau (p-Tau) are considered the two critical biomarkers in AD brains (Congdon and Sigurdsson, 2018). It has been reported that oxidative stress is a conspicuous cause of Aβ aggravation and p-Tau formation for AD development (Jiang et al., 2018).

Aβ is produced from amyloid-β precursor protein (APP) via the amyloidogenic pathway. APP is widely present in various tissues, with its highest expression in the synapses of neurons. APP is synthesized in the endoplasmic reticulum, then transported to the Golgi apparatus, where it is mistakenly cleaved by β-secretase (BACE1) into β-N-terminal and β-C-terminal fragments. The N-terminal transmembrane region of the β-C-terminal fragment is further hydrolyzed by γ-secretase on the cell membrane to release an Aβ peptide (consisting of 39–43 amino acids), and the peptide further aggregates to form Aβ plaques (Hefter et al., 2020). In physiological conditions, the non-amyloidogenic pathway of APP cleavage, which is hydrolyzed by α- and γ-secretase, generates soluble α-APP, p3, and α-C terminal fragments (Dar and Glazner, 2020). In the brains of AD patients, the amyloidogenic pathway appears to be increased.

Presenilin 1 (PSEN1) is one of the subunits of γ-secretase, the mutation of which is highly expressed in most AD patients and considered to be one of the main genetic factors of familial AD (Kabir et al., 2020). PSEN1 mutation may influence the expression of both β-secretase and other subunits of γ-secretase, including PSEN2, nicastrin, presenilin enhancer 2 (PEN2), and anterior pharynx-defective 1 (APH1), thus enhancing the maturation of Aβ (Li et al., 2011; Liu et al., 2019). Oxidative stress in the brain, which may upregulate β-secretase expression and increase the amyloidogenic cleavage of APP, also promotes AD (Sharma et al., 2019). Some natural ingredients, such as folic acid and berberine, have been shown to inhibit the deposition of Aβ by regulating the expression of b- and γ-secretase, which has become a strategy to alleviate the progression of AD (Tian et al., 2016; Cai et al., 2018).

Moreover, extracellular Aβ plaques interfere with the normal growth of neurons and further enhance oxidative stress, represented by overproduction of reactive oxygen species (ROS) (Sultana et al., 2009). Elevated oxidative stress leads to increased DNA, RNA, lipid, and protein oxidation in the brains of AD patients, which causes neuronal cell death (Halliwell, 2006). Antioxidant factors, such as glutathione peroxidase (GSH-PX) and superoxide dismutase (SOD), have been considered therapeutic targets that inhibit AD pathogenesis (Chen et al., 2019). In addition, ROS and Aβ accumulation in the AD brain can potently activate glial cells to release inflammatory cytokines and cause severe brain inflammation (Houtman et al., 2019). The inflammatory cytokines, including interleukin 1β (IL-1β) in the brain, can increase phosphorylation of Tau and make it dissociate from neuronal synapses (Graham et al., 2017). In a physiological state, Tau maintains the stability of neurons and synapses. However, in the AD condition, dissociated p-Tau forms NFTs in neurons, causing synapse damage, which obstructs transmission of neural signals and further aggravates cognitive dysfunction (Hoppe et al., 2010). Therefore, blocking oxidative stress could be an effective strategy to slow the AD process.

Cerebrovascular function is commonly affected in AD patients (Fouda et al., 2019). The clearance of Aβ could be impaired due to cerebrovascular damage in the early stage, which further accelerates AD (Gupta and Iadecola, 2015). In addition, cerebrovascular damage causes insufficient supply of oxygen and glucose in neurons and glial cells, resulting in decreased ATP production and enhanced oxidative stress (Merlini et al., 2011). Long-term chronic hypoxia ultimately causes irreversible structural damage to cells and tissues of the central nervous system (Daulatzai, 2017).

The LongShengZhi capsule (LSZ), a well-known traditional Chinese herbal medicine, has long been prescribed to patients with cardiovascular and cerebrovascular diseases in China. It offers a potent protection against atherosclerotic lesions, ischemic brain damage, and vascular dementia (Ma et al., 2019; Yang et al., 2020). LSZ is made from 12 traditional Chinese medicines, including *Hirudo* (Shuizhi), *Pheretima* (Dilong), *Astmgali Radix* (Huangqi), *Chuanxiong Rhizoma* (Chuanxiong), *Angelicae Sinensis Radix* (Danggui), *Carthami Flos* (Honghua), *Persicae Semen* (Taoren), *Paeoniae Radix Rubra* (Chishao), *Aucklandiae Radix* (Muxiang), *Acori Tatarinowii Rhizoma* (Shichangpu), *Talxilli Herba* (Sangjisheng), and extract of *Acanthopanax Senticosus* (Ciwujia), as shown in Table 1 (Wei et al., 2019). The 19 main active ingredients in LSZ are listed in Table 2. Many of them have clear protective effects on the central nervous system. For example, *Astmgali Radix* can reduce neuroinflammation and cognitive impairment (Huang et al., 2017). *Carthami Flos* seed can attenuate memory impairment induced by scopolamine in mice by ameliorating cholinergic dysfunction and oxidative stress (Kim et al., 2019). *Acanthopanax senticosus* extract also has a neuroprotective effect on nervous system diseases (Li et al., 2014). Moreover, bioactive components contained in LSZ, such as paeoniflorin, isoflavone glucoside, and syringin, have been reported to inhibit oxidative stress to protect neurons (Ding et al., 2013; Zhong et al., 2015).

**TABLE 1:** Main ingredients in LSZ.

Ingredients	Local name	Part used	Biological properties
*Hirudo*	Shuizhi	Dried body	Anti-thrombosis; protection against cerebral ischemia reperfusion injury
*Pheretima*	Dilong	Dried body	Anti-cerebral thrombosis; anti-inflammation
*Astmgali Radix*	Huangqi	Root	Neuroprotection; cognition improvement; glucose and lipid metabolism improvement
*Chuanxiong Rhizoma*	Chuanxiong	Rhizome	Neuroprotection; anti-neuroinflammation; cerebral vascular function improvement
*Angelicae Sinensis Radix*	Danggui	Root	Blood vessel protection; neuroprotection; anti-neuroinflammation;
*Carthami Flos*	Honghua	Flower	Cerebral ischemia improvement; anti-neuroinflammation; angiogenesis promotion
*Persicae Semen*	Taoren	Dried seed	Anticoagulant; neurotrophic
*Paeoniae Radix Rubra*	Chishao	Root	Microcirculation improvement; Blood vessel dilation; myocardial ischemia prevention; anti-thrombosis
*Aucklandiae Radix*	Muxiang	Root	Gastrointestinal function improvement; vascular tension regulation
*Acori Tatarinowii Rhizoma*	Shichangpu	Root	Antidepressant; neuroprotection; inhibiting glial inflammation; cognition improvement
*Talxilli Herba*	Sangjisheng	Stem	Anti-oxidation; anti-inflammation; anti-cancer
extract of *Acanthopanax Senticosus*	Ciwujia	Stem	Anti-anxiety; neuroprotection; cerebral vascular function protection

**TABLE 2:** Main active components in LSZ-EES.

Component	Proportion (∼mg⋅g^–1^)	Component	Proportion (∼mg⋅g^–1^)
Paeoniflorin	3.9	Senkyunolide I	0.05
Calycosin-7-glucoside	0.75	Dehydrocostus lactone	0.03
Amygdalin	0.7	Calycosin	0.03
Hydroxysafflor yellow A	0.49	Ligustilide	0.01
Syringin	0.25	Senkyunolide A	0.01
Ferulic acid	0.15	Oxypaeoniflora	0.01
Eleutheroside E	0.15	Senkyunolide H	<0.01
Isofraxidin	0.13	Ligustrazine	<0.01
Benzoylpaeoniflorin	0.11	3-n-Butylphthalide	<0.01
Astragaloside A	0.1		

Our previous studies have shown that LSZ can reduce thrombosis by inhibiting platelet adhesion through reducing oxidative stress and vascular inflammation in the aorta of atherosclerotic mice (Li et al., 2019; Ma et al., 2019). Moreover, we have demonstrated its strong antioxidant role in heart failure (Xu et al., 2020). These findings suggest a potential role of LSZ in AD treatment. Therefore, in this study, we used human APP and mutant presenilin 1 double transgenic (APP/PS1) mice and HT-22 cells to determine the anti-AD-like pathology effect of LSZ.

## Materials and Methods

### Chemicals and Antibodies

LongShengZhi Capsule (CAT# A14001035472) was provided primarily by the Buchang Pharmaceutical Co. Ltd. (Xi’an, Shaanxi, China). The LSZ ethanol extract solution (LSZ-EES) in PBS was prepared as follows (Li et al., 2019). Bovine serum albumin (BSA), 3-(4,5-dimethylthiazol-2-yl)-2,5-diphenyltetrazolium bromide (MTT), L-glutamic acid (L-Glu), and thioflavin S were purchased from Sigma Aldrich (St. Louis, MO, United States). 2’7’-Dichlorodihydrofluorescein diacetate (DCFH-DA) was purchased from Meilun Biotechnology (Dalian, Liaoning, China). A cocktail of protease inhibitors, PMSF, and enhanced chemiluminescence (ECL) kits were purchased from Millipore (Darmstadt, Germany). Bromphenol blue, eosin Y, heparin, triton X-100, and sodium dodecyl sulfate (SDS) were purchased from the Solarbio (Beijing, China). 4’,6-Diamidino-2-phenylindole (DAPI) was purchased from Santa Cruz Biotechnology (Paso Robles, CA, United States). Hematoxylin staining solution, phosphate buffer saline (PBS), BCA protein assay kit, and 4% polyformaldehyde were purchased from Biosharp (Hefei, Anhui, China). Terminal deoxynucleotidyl transferase (TdT)-mediated dUTP nick end labeling (TUNEL) assay kit was purchased from KeyGen Biotech (Nanjing, Jiangsu, China). FAS siRNA kit (CAT# siB13118141431) was purchased from RiboBio Co., Ltd. (Guangzhou, Guangdong, China). Lipofectamine^TM^ RNAiMAX (CAT# 13778030) was purchased from Thermo Scientific (Waltham, MA, United States). Total RNApure reagent (Tri-zol, ZP401-2) was purchased from Zomen Biotechnology Co. Ltd. (Beijing, China). The HiScript II Q Select RT SuperMix for qPCR and AceQ SYBR qPCR Master Mix were purchased from Vazyme (Piscataway, NJ, United States). Annexin V-PE analysis kit, goat anti-rabbit lgG (H + L)-HRP (CAT# LK2001, 1:10000), and goat anti-mouse lgG (H + L)-HRP (CAT# LK2003, 1:10000) were purchased from Sungene Biotech (Tianjin, China). Rabbit anti-PSEN1 (CAT# 20434-1-AP, 1:100), Tau (CAT# 10274-1-AP, 1:1000), Bax (CAT# 50599-2-lg, 1:8000), Bcl-2 (CAT# 12789-1-AP, 1:1000), Bad (CAT# 10435-1-AP, 1:2000), Iba-1 (CAT# 10904-1-AP, 1:300), and FITC-goat anti-mouse IgG (H + L) (CAT# SA00003-1, 1:100), FITC-goat anti-rabbit IgG (H + L) (CAT# SA00003-2, 1:100), and TRITC-goat anti-rabbit IgG (H + L) (CAT# SA00007-2, 1:100) antibodies were purchased from Proteintech Group, Inc. (Chicago, IL, United States). Rabbit anti-APP (CAT# AF6084, 1:300) antibody was purchased from Affinity Biosciences (Changzhou, Jiangsu, China). Rabbit anti-phospho-MAPT-S404 (p-Tau, CAT# AP0170, 1:1000 for WB; 1:100 for IF), IL-1β (CAT# A17361, 1:1000 for WB; 1:100 for IF), NF-κB (CAT# A16271, 1:1000 for WB; 1:100 for IF), β-actin (CAT# AC026, 1:200000), and mouse anti-GAPDH (CAT# AC033, 1:200000) antibodies were purchased from Abclonal (Wuhan, Hubei, China).

### Cell Culture

HT-22 cells, a mice hippocampal neuronal cell line (CAT# CP-H042), were purchased from Procell (Wuhan, Hubei, China) and cultured in Dulbecco’s Modified Eagle’s Medium (DMEM, Biological Industries, Kibbutz Beit Haemek, Israel) containing 10% fetal bovine serum (FBS, AusGeneX, Brisbane, Australia) as well as 50 μg/mL streptomycin/penicillin (Hyclone, Logan, UT, United States) at 37°C in an atmosphere of 5% CO_2_. Cells at ∼90% confluence were treated in serum-free medium.

### Cell Viability Assay

Cell viability was detected using MTT assay. In brief, HT-22 cells were plated into 96-well plates (∼10^4^ cells/well) and were treated as indicated. After treatment, the culture medium was removed, and cells were added to MTT (0.5 mg/mL) and incubated for 4 h at 37°C. After washing with PBS 3 times and incubating in DMSO (150 μL/well) for 15 min, the optical density at a wavelength of 550 nm was read by a microplate reader (BioTek Instruments, Winooski, VT, United States). The mean of absorbance was expressed as fold changes compared to control samples.

### Cell Death Assay

HT-22 cells were seeded in 6-well plates and pre-treated with LSZ-EES for 3 h followed by co-treatment with L-Glu for another 18 h. Then the cells were lifted by trypsin (without EDTA) and washed with cold PBS twice. After incubation with annexin V-PE and 7-aminoactinomycin D (7-AAD) working solution in darkness at room temperature for 20 min, the fluorescence intensity was measured by the fluorescence-activated cell sorting (FACS) assay using a CytoFLEX (Beckman, Brea, CA, United States).

### ROS Determination

HT-22 cells were plated in 96-well plates or 6-well plates and pre-treated with LSZ-EES for 3 h, followed by co-treatment with L-Glu for another 18 h. After treatment and removal of the treatment medium, cells were incubated with DCFH-DA (10 μM) working solution for 20 min at 37°C in darkness. After being washed with PBS 3 times, samples in 96-well plates were measured on a microplate reader at 504 nm (excitation) and 529 nm (emission), while the fluorescence images in 6-well plates were captured with a fluorescence microscope (Leica, Wetzlar, Germany).

### siRNA Transfection

HT-22 cells in 6-well plates at ∼40% confluence were transfected with FAS siRNA (siFAS, 50 nM/well) or the corresponding negative control siRNA (siCtrl, 50 nM/well) using Lipofectamine^TM^ RNAiMAX in serum-free medium for 24 h, then incubated in complete DMEM medium for another 24 h, followed by the treatment indicated.

### Animals

The experiment was conducted with the approval of the Institution Animal Ethics Committee of Hefei University of Technology (HFUT20190116001). All animal experiments were performed in compliance with the guidelines published in the National Institutes of Health (NIH) Guide for the Care and Use of Laboratory Animals. We used male mice in this study, which is widely accepted in AD-related studies, to avoid the hormonal changes, including estrogen, in female mice in AD progress. In addition, all animals were randomized before received treatment. The animals were maintained at a temperature of 24 ± 2°C with a 12-h light/dark cycle and checked daily during the experiment for food intake, water consumption, and bodyweight.

Both male C57BL/6J wild-type mice and APP/PS1 mice (∼2 months old, ∼20 g) without backcrossing were purchased from the Model Animal Research Center of Nanjing University (Nanjing, Jiangsu, China). All the animals were free to access food and drinking water. C57BL/6J wild-type mice fed normal chow were used as the normal control (Group 1, WT, 12 mice/group). APP/PS1 mice were randomly divided into three groups (12 mice/group) and received the following treatment. Group 2 (AC), APP/PS1 mice were fed normal chow. Group 3 (ALN), APP/PS1 mice were fed normal chow containing LSZ at a normal dose (850 mg/100 g food, based on clinical dosage). Group 4 (ALH), APP/PS1 mice were fed normal chow containing LSZ at a high dose (2000 mg/100 g food). The treatment lasted for 7 months as the pathological changes in the brains of APP/PS1 mice at 9 months old are severe enough to be detected.

### Morris Water Maze (MWM) Test

The MWM video tracking analysis system (1056001, Beijing ZS Dichuang Technology Development Co., Ltd., Beijing, China) was used to assess spatial learning and reference memory (Vorhees and Williams, 2006). A circular pool was divided into four quadrants and filled with water (22 ± 2°C). A target platform was fixed and immersed at a depth of 2 cm in the middle of the first quadrant. Titanium dioxide was added to the pool to make the platform invisible. A tracking camera was placed above the pool to record the trajectories of the mice. The MWM experiment consisting of a spatial probe test and a hidden platform trial was performed over the next 6 consecutive days as follows (Nunez, 2008). Briefly, in the acquisition training phase, the mouse was placed into the water facing the wall of the pool and swam freely. The length of time it took the mouse to find the underwater platform was recorded. If the mouse did not find the platform within 60 s, we would guide it to the platform and allow it to remain there for 10 s. After training, the animals were removed, dried, and then returned to the cage. Each mouse was trained 4 times a day for 5 consecutive days, entering the water from a different quadrant each time. On the second day after the final acquisition training (day 6), the platform was removed to perform the 60-s probe trial. The mouse was placed in water in the opposite quadrant from where the original platform was located. The trajectories, times of passing through the target quadrant or the original platform, and escape latency that the mice arrived at the hidden platform were recorded.

### Y Maze (YM) Test

The YM video tracking analysis system (1056006, Beijing ZS Dichuang Technology Development Co., Ltd., Beijing, China) was used to test the discriminatory working memory and spatial exploration ability of mice (Oboh et al., 2020). Three arms of the YM were randomly defined as the new arm, the starting arm, and the third arm. Mice were placed into the maze at the end of the starting arm and allowed to explore the maze for 3 min. A camera was fixed 1.5 m above the maze to record the trajectory, distance, and the time and length of time it took the mice to cross the new arm.

### Brain Section Preparation

For histological examination, six mice per group were selected randomly and anesthetized with 2% sodium pentobarbital. Mice were then perfused with PBS and 4% paraformaldehyde, followed by collection of whole brain tissue. The brain tissue was quickly frozen with dry ice and then fixed in 4% paraformaldehyde for 24 h at 4°C. Afterward, the brains were dissected and separated into two hemispheres: one for paraffin section preparation and the other for frozen section preparation. Hemispheres for paraffin sections were dehydrated using a HistoPearl automatic dehydrator (Leica, Wetzlar, Germany) and embedded with paraffin. Hemispheres for frozen sections were dehydrated with 15, 20, and 30% sucrose solutions in turn, each for 24 h, and then embedded in optical cutting temperature compound (OCT) and stored at –80°C until sectioning.

For protein and RNA extraction, another six mice per group were euthanatized, and brain tissue was immediately dissected. The cortex and hippocampus were separated and stored at –80°C until processing.

### Immunofluorescence Staining and Thioflavin S Staining

The paraffin-embedded brain was cut into 5-μm sections. Expression of p-Tau, PSEN1, NF-κB, IL-1β was detected as follows (Chen et al., 2014). The frozen brain in OCT was cut into 20-μm sections to determine Iba-1 and Aβ plaques. Aβ plaques were detected by co-staining with APP antibody and thioflavin S. Briefly, the sections were blocked with 3% BSA for 1 h at room temperature and then incubated with diluted primary antibody overnight at 4°C. The sections were washed with PBS 3 times, for 10 min each time, and then incubated with FITC-goat anti-rabbit IgG or TRITC-goat anti-rabbit IgG secondary antibodies for 1 h at room temperature. Samples incubated without primary antibody were used as the negative control (NC) to illustrate the specificity of the primary antibody. The sections were washed with PBS for 3 × 10 min and then stained with DAPI solution for nucleus. For Aβ plaque determination, sections were washed with PBS for 3 × 5 min after DAPI staining. The sections were incubated with 0.5% thioflavin S solution for 10 min at room temperature and then washed with 70% alcohol for 15 min and then with PBS for 5 min. The sections were covered with coverslips and kept in the dark until dry. Images were obtained using a ZEISS Scope A1 fluorescence microscope. The ratio of mean of fluorescence intensity (MFI) to cell number or Iba-1-positive cells to cell number were assessed using Photoshop by a technician who was blind to the experiment.

### TUNEL Staining

The paraffin-embedded brain was cut into 5-μm sections to detect cell death in brain tissues using a TUNEL assay kit as follows (Ma et al., 2019). Images were observed with a ZEISS Scope A1 fluorescence microscope. The number of TUNEL-positive cells in more than three random fields was counted and quantitated as per mm^2^ by a technician who was blind to the experiment and expressed as fold of the WT group.

### Quantitative Real Time PCR (qRT-PCR) and Western Blot

After treatment, the total RNA was extracted from HT-22 cells or 30-mg brain tissues using Trizol as follows (Chen et al., 2014). cDNA was synthesized with the same amount of total RNA from each sample with HiScript II Q Select RT SuperMix (+gDNA wiper). qRT-PCR was then performed using the AceQ SYBR qPCR Master Mix reverse transcriptase kit and the primers listed in Table 3 on LightCycler96 (Roche, Mannheim, Germany). The mRNA expression was normalized by β-actin mRNA in the corresponding samples.

**TABLE 3:** Sequences of primers for qRT-PCR assay.

Gene	Forward sequences	Reverse sequences
β-actin	ATGGAGGGGAATACAGCCC	TTCTTTGCAGCTCCTTCGTT
IL-1β	GACCTTCCAGGATGAGGACA	AGCTCATATGGGTCCGACAG
IL-10	GCTCTTACTGACTGGCATGAG	CGCAGCTCTAGGAGCATGTG
IL-6	GAGGATACCACTCCCAACAGACC	AAGTGCATCATCGTTGTTCATACA
TNF	CGTCGTAGCAAACCACCAAG	TTGAAGAGAACCTGGGAGTAGACA
SOD1	GCCTTGTGTATTGTCCCCAT	ACCATCCACTTCGAGCAGAA
SOD2	AGACACGGCTGTCAGCTTCT	CTGGACAAACCTGAGCCCTA
GSH-PX	CAATGTAAAATTGGGCTCGAA	GTTTCCCGTGCAATCAGTTC
Bcl-2	CTTTGAGTTCGGTGGGGTCA	GCCCAGACTCATTCAACCAGA
Bad	CCCTTAGAACTGGAGGGAGGA	GTCGCATCTGTGTTGCAGTG
Bax	AGGATGCGTCCACCAAGAAG	CTTGGATCCAGACAAGCAGC
Bcl-xL	CTACCAGGTCGCATGATCCC	CCCGGTTGCTCTGAGACATT
FAS	TGCTTGCTGGCTCACAGTTA	GAATCACTCCAACGGGCTGA
BACE1	GCTGGGAGCTGGATTATGGT	CTATCCGAGCCCGTAGCTTT
APH1a	TATCCACTGCCCATGACTGA	GCAGATTCAAACCCACCAGT
PEN2	TGTTGTGAAGTCGGGAGCAC	AGTACTTCCGGCACAGGTTC

*IL-1β/6/10, interleukin 1β, 6 or 10; TNF, tumor necrosis factor; SOD1, superoxide dismutase 1; SOD2, superoxide dismutase 2; GSH-PX, glutathione peroxidase; Bcl-2, B-cell lymphoma 2; Bad, Bcl-2 associated agonist of cell death; Bax, Bcl-2 associated X protein; Bcl-xL, B-cell lymphoma-extra large; FAS, Fas cell surface death receptor; BACE1, beta-site APP-cleaving enzyme 1; APH1a, anterior pharynxdefective 1a; PEN2, presenilin enhancer 2.*

After treatment, HT-22 cells or 30-mg brain tissues were lysed or grated with lysis buffer (Chen et al., 2012). Protein concentration was determined using the BCA protein assay kit. The same amount of protein (50 μg) from each sample was used to determine protein expression of Tau, p-Tau, FAS, Bcl-2, Bad, Bax, NF-κB, IL-1β, p53, GAPDH, or β-actin by Western blot as described (Sun et al., 2017). The signals were detected on Chemiscope 3000 mini (Qinxiang, Shanghai, China) and the band density was quantified using Photoshop.

### Statistical Analysis

Based on a survey of data from published research or preliminary studies, we performed *in vitro* studies at least five independent times and decided on the sample size for the *in vivo* studies. All data were analyzed by a technician who was blind to the experiment design and expressed as mean ± standard deviation (SD). Graph Pad Prism 7.0 was used for statistical analysis. Two-way ANOVA was used to assess significance in ROS and qRT-PCR of the *in vitro* study. One-way ANOVA was used on the other data to assess significance. The Shapiro-Wilk normality test, White test, and Brown-Forsythe test were conducted on the data to evaluate if the values came from a Gaussian distribution (Supplementary Material). The differences were considered significant at *p* < 0.05.

## Results

### LSZ-EES Rescues L-Glu-Induced HT-22 Cell Death and Oxidative Stress

**FIGURE 1:**
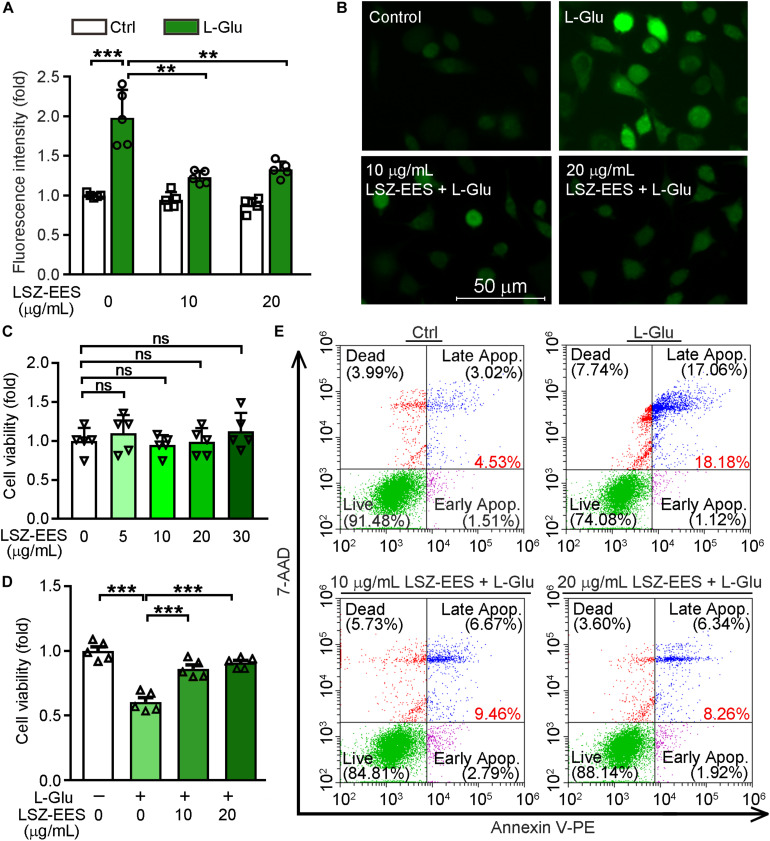
LongShengZhi Capsule Enhances HT-22 Cell Survival by Reducing L-Glu-Induced Oxidative Stress. **(A,B,D,E)** HT-22 cells were pre-treated with LSZ-EES (0, 10, and 20 μg/mL) for 3 h, then co-treated with 20 mM L-Glu for another 18 h; **(C)** HT-22 cells were treated with LSZ-EES at the indicated concentrations for 18 h. ROS levels were detected with DCFH-DA (**A,B**; scale bar: 50 μm). Cell viability was determined by MTT **(C,D)**, and cell death was determined by FACS **(E)**. **p < 0.01; ***p < 0.001; ns, not significant (*n* = 5).

LongShengZhi Capsule has been reported to reduce thrombosis by inhibiting ROS production in human umbilical vein endothelial cells (Li et al., 2019). Thus, we speculated that LSZ may have similar antioxidant effects on neuronal cells. L-Glu is an excitatory neurotransmitter and induces neuronal death by activating oxidative stress (Kritis et al., 2015). A close association between ROS levels and synaptic dysfunctions has been reported (Li et al., 2017). To determine the protective effects of LSZ against neuronal damage, we initially pre-treated HT-22 cells, a neuronal cell line, with LSZ-EES for 3 h. Cells were then treated with L-Glu to induce HT-22 cell death. We found L-Glu exposure increased ROS production to ∼two-fold (two-way ANOVA, *F*_(__2_, _24__)_ = 21.11, *p* = 0.0002) of that in the control group (Ctrl). However, the increase was substantially blocked by LSZ-EES co-treatment (two-way ANOVA, 39%, *F*_(__2_, _24__)_ = 12.61, *p* = 0.0015 for 10 μg/mL LSZ-EES; 33%, *F*_(__2_, _24__)_ = 12.61, *p* = 0.0017 for 20 μg/mL LSZ-EES) (Figures 1A,B).

Next, we determined the effect of LSZ on cell viability using the MTT method. As expected, LSZ-EES alone had little effect on cell viability, indicating its high degree of safety to cells (*F*_(__4_, _20__)_ = 0.756, *p* > 0.05) (Figure 1C). In contrast, L-Glu clearly reduced cell viability by ∼43% (*F*_(__3_, _20__)_ = 33.39, *p* = 0.0003), but the reduction was reversed by LSZ-EES treatment (*F*_(__3_, _20__)_ = 33.39, *p* = 0.0009 for 10 μg/mL LSZ-EES, *p* = 0.0007 for 20 μg/mL LSZ-EES) (Figure 1D). In addition, the results of the flow cytometry experiment show that L-Glu increased the percentage of cell death at early and late stages from ∼4.5% in the control group to ∼18.2%, but the number was significantly reduced to ∼9.5 or ∼8.3% by LSZ-EES treatment at 10 or 20 μg/mL (Figure 1E). These results suggest that LSZ-EES can enhance HT-22 cell viability, which is related to the reduction of L-Glu-induced oxidative stress.

**FIGURE 2:**
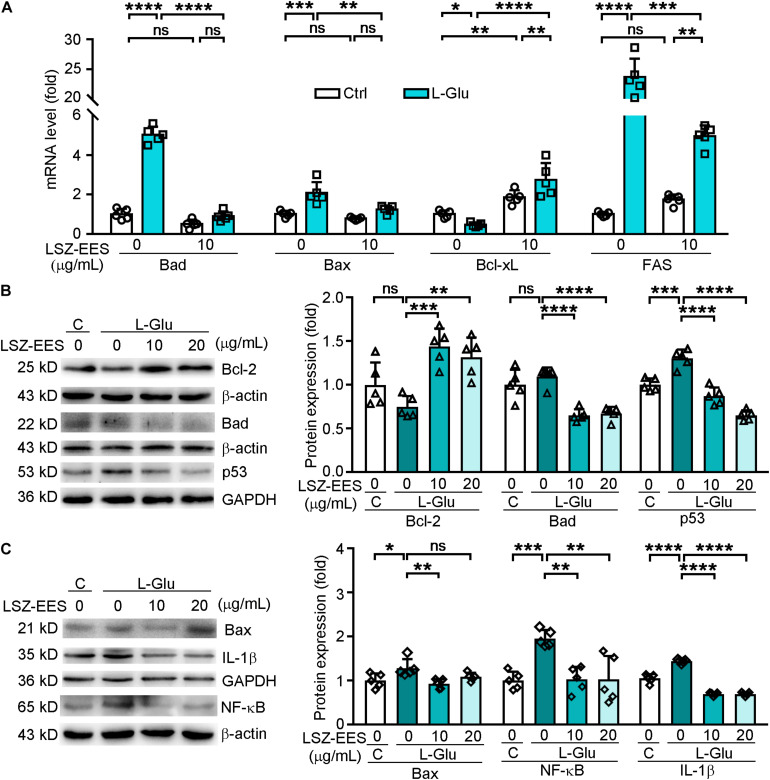
LongShengZhi Capsule Antagonizes L-Glu-induced Expression of Apoptosis-Associated Genes and Inflammatory Cytokines in HT-22 Cells. A: HT-22 cells were pre-treated with LSZ-EES (10 μg/mL) for 3 h, followed by co-treatment with 20 mM L-Glu for another 18 h, or LSZ-EES alone for 21 h; **(B,C)** HT-22 cells were pre-treated with LSZ-EES at the indicated concentrations for 3 h and then co-treated with 20 mM L-Glu for an additional 18 h. Expression of Bad, Bax, Bcl-xL, and FAS mRNA was determined by qRT-PCR **(A)**. Expression of Bcl-2, Bad, p53, Bax, IL-1β, and NF-κB protein was detected by Western blot with quantitative analysis of band density and normalization to β-actin or GAPDH **(B,C)**. *p < 0.05; **p < 0.01; ***p < 0.001; ****p < 0.0001, ns, not significant (*n* = 5).

Reactive oxygen species is mainly produced by mitochondria, so the neuroprotection of HT-22 cells by LSZ-EES against L-Glu-induced neurotoxicity may be related to its effect on expression of mitochondrial apoptotic genes. The Bcl-2 family is among the essential factors regulating mitochondrial membrane permeability and the mitochondrial apoptosis pathway (Rong and Distelhorst, 2008). Our results show that L-Glu reduced the anti-apoptotic gene Bcl-xL mRNA level (two-way ANOVA, *F*_(__1_, _16__)_ = 29.14, *p* = 0.0403) and Bcl-2 protein expression (*F*_(__3_, _16__)_ = 11.36, *p* = 0.198), while inducing pro-apoptotic genes, Bax and Bad expression, at protein (*F*_(__3_, _16__)_ = 6.192, *p* = 0.0152 for Bax; *F*_(__3_, _16__)_ = 21.74, *p* = 0.6742 for Bad) or mRNA levels (two-way ANOVA, *F*_(__1_, _16__)_ = 4.902, *p* = 0.0003 for Bax; *F*_(__1_, _16__)_ = 188.1, *p* < 0.0001 for Bad). However, the effect of L-Glu on expression of those genes was significantly reversed by LSZ-EES treatment (Figures 2A-C), suggesting the anti-mitochondria-associated apoptotic effect of LSZ-EES.

FAS is a pro-apoptotic gene located on the membrane surface and an upstream molecule of the Bcl-2 family (Strasser et al., 2009). Once activated, FAS transmits stimulus signals into the membrane, and its intra-membrane domain leads to programmed cell death (Kaufmann et al., 2012). To determine if LSZ can block the death signal induced by L-Glu from extra-cellular to intra-cellular, we detected expression of FAS and p53. As shown in Figure 2A, the FAS mRNA level was significantly activated by L-Glu (two-way ANOVA, *F*_(__2_, _24__)_ = 186.6, *p* < 0.0001), but the activation was antagonized by LSZ-EES treatment (two-way ANOVA, *F*_(__2_, _24__)_ = 186.6, *p* = 0.0009). Furthermore, LSZ-EES treatment also inhibited L-Glu-induced p53 protein expression, a pro-apoptotic gene responsible for DNA damage (*F*_(__3_, _16__)_ = 51.29, *p* < 0.001) (Figure 2B). These results suggest LSZ-EES may enhance neuron survival by regulating FAS/Bcl-2/p53 expression.

Therefore, we transfected HT-22 cells with FAS siRNA to indicate the mechanisms of LSZ-EES in regulating neuronal death. As shown in Supplementary Figure 1, in cells not transfected with FAS siRNA, LSZ-EES significantly up-regulated protein expression of Bcl-2 (two-way ANOVA, *F*_(__3_, _32__)_ = 67.63, *p* < 0.0001) and mRNA expression of Bcl-xL (two-way ANOVA, *F*_(__3_, _32__)_ = 27.99, *p* < 0.0001) and down-regulated protein and mRNA expression of Bax (two-way ANOVA, *F*_(__3_, _32__)_ = 90.43, *p* < 0.0001 for protein; *F*_(__3_, _32__)_ = 22.26, *p* < 0.0001 for mRNA) and mRNA expression of Bad (two-way ANOVA, *F*_(__3_, _32__)_ = 12.12, *p* < 0.0001), while in transfected FAS siRNA cells, compared with the L-Glu-exposed group, LSZ-EES pre-treatment failed to regulate Bcl-2 (two-way ANOVA, *F*_(__3_, _32__)_ = 67.63, *p* > 0.05), Bcl-xL (two-way ANOVA, *F*_(__3_, _32__)_ = 27.99, *p* > 0.05), Bax (two-way ANOVA, protein: *F*_(__3_, _32__)_ = 90.43, *p* > 0.05; mRNA: *F*_(__3_, _32__)_ = 22.26, *p* > 0.05), and Bad (two-way ANOVA, *F*_(__3_, _32__)_ = 12.12, *p* > 0.05) expression, indicating that silencing of FAS eliminated the regulation of apoptotic factors by LSZ-EES. Taken together, these data indicate that LSZ-EES reduces L-Glu-induced ROS production and inhibits neuronal cell death, at least in part, through the FAS/Bcl-2/p53 pathway.

Elevated ROS production is often accompanied by high levels of inflammation in AD patients (Cooney et al., 2014). NF-κB has been shown to play a role in mediating production of inflammatory cytokines in the AD process (Sandireddy et al., 2014). We found that treatment of HT-22 cells with L-Glu increased protein levels of IL-1β (*F*_(__3_, _16__)_ = 170.3, *p* < 0.0001) and NF-κB (*F*_(__3_, _16__)_ = 9.999, *p* = 0.0010), which were blocked by LSZ-EES treatment (*F*_(__3_, _16__)_ = 170.3, *p* < 0.0001 for IL-1β; *F*_(__3_, _16__)_ = 9.999, *p* = 0.0013 for NF-κB) (Figure 2C). These results show that LSZ-EES may prevent neurons from inflammation by reducing oxidative stress.

### LSZ Improves Cognitive Function in APP/PS1 Mice

**FIGURE 3:**
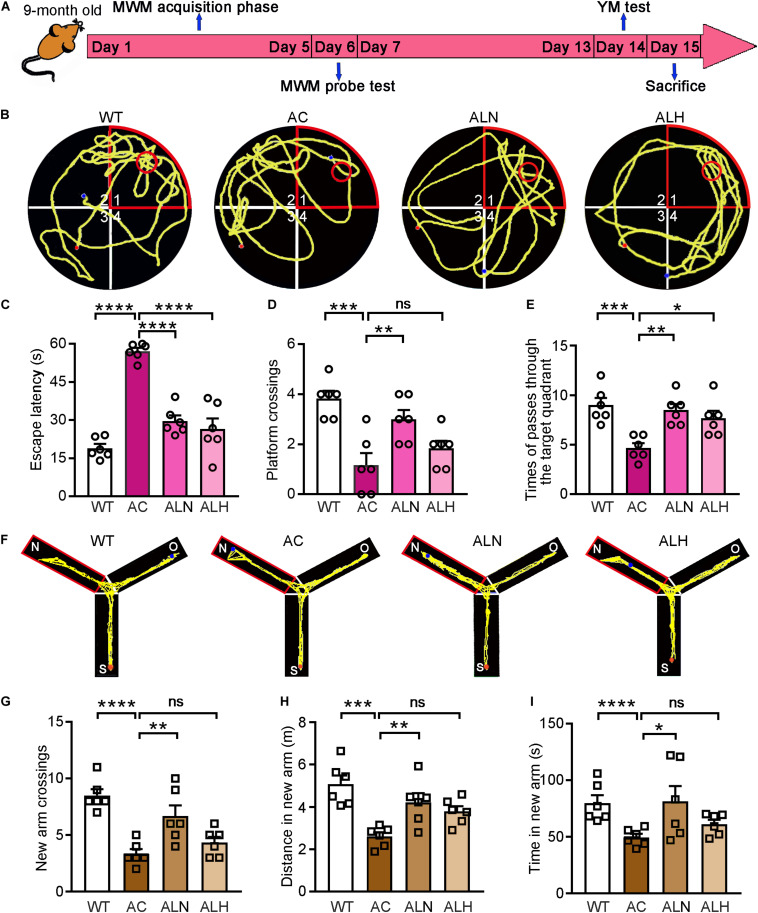
LongShengZhi Capsule Improves Cognitive Functions in APP/PS1 Mice. **(A)** The design of behavioral experiments; **(B–E)** MWM test was conducted on mice. Record of motion trajectory **(B)**, escape latency **(C)**, platform crossings in the target quadrant **(D)**, and times of passing through the target quadrant **(E)** were recorded. The red sector and circle represent the target quadrant and the target platform, respectively, and the yellow curve represents the behavioral track of the mice; **(F–I)** YM test was conducted on mice. Motion behavior track **(F)**, times of crossing new arm **(G)**, distance **(H)**, and time **(I)** in the new arm were recorded. *p < 0.05; **p < 0.01; ***p < 0.001; ****p < 0.0001; ns, not significant (*n* = 6).

Next, we used APP/PS1 mice to determine the cognitive protection of LSZ in the development of AD-like pathology. The 2-month-old APP/PS1 mice were divided into three groups. The animals were fed normal chow, normal chow containing a normal dose (850 mg/100 g food), or a high dose (2000 mg/100 g food) of LSZ, respectively. The age-matched WT mice fed normal chow were used as the normal control. After 7 months of treatment, behavioral experiments, including MWM and YM, were conducted to detect spatial memorizing (Figure 3A). In the MWM test, the AC group required significantly more time than the WT group to reach the platform (*F*_(__3_, _20__)_ = 41.9, *p* < 0.0001), while LSZ treatment significantly reduced the escape latency (*F*_(__3_, _20__)_ = 41.9, *p* < 0.0001) (Figures 3B,C). Accordingly, the times for crossing the target platform and quadrant decreased by ∼75% and ∼46%, respectively, in the AC group compared to the WT group (*F*_(__3_, _20__)_ = 10.29, *p* = 0.0002 for crossing the target platform, *F*_(__3_, _20__)_ = 8.663, *p* = 0.0005 for crossing the target quadrant), while LSZ treatment reversed these parameters by ∼157% for crossing the target platform (AC vs. ALN, *F*_(__3_, _20__)_ = 10.29, *p* = 0.0068), and 82% (*F*_(__3_, _20__)_ = 8.663, *p* = 0.0016) and ∼64% (*F*_(__3_, _20__)_ = 8.663, *p* = 0.0129) for crossing the target quadrant (Figures 3D,E).

To eliminate the mutual interference between the two behavioral experiments, the YM test was performed one week later after the MWM test. Our results showed that WT mice had more trajectories in the new arm than the other two arms, showing the normal exploring and learning behavior. In contrast, decreased exploring in the new arm was observed in the AC group but not in the LSZ-treated groups (Figure 3F). In addition, compared with the AC group, LSZ treatment increased the relative number (to ∼1.6-fold vs. ∼1.1-fold), distance (to ∼1.6-fold vs. ∼1.5-fold), and time (to ∼1.6-fold vs. ∼1.2-fold) of exploring in the new arm with a greater effect by LSZ at the normal dose (*F*_(__3_, _20__)_ = 13.13, *p* = 0.0046 for number; *F*_(__3_, _20__)_ = 9.215, *p* = 0.0091 for distance; *F*_(__3_, _20__)_ = 15.89, *p* = 0.0217 for time) (Figures 3G-I), suggesting that LSZ can enhance the working memory in APP/PS1 mice. Taken together, the results in Figure 3 indicate that LSZ can repair the cognitive impairment in APP/PS1 mice.

### LSZ Attenuates Cell Death and Enhances Antioxidant Enzyme Expression in the APP/PS1 Mouse Brain

**FIGURE 4:**
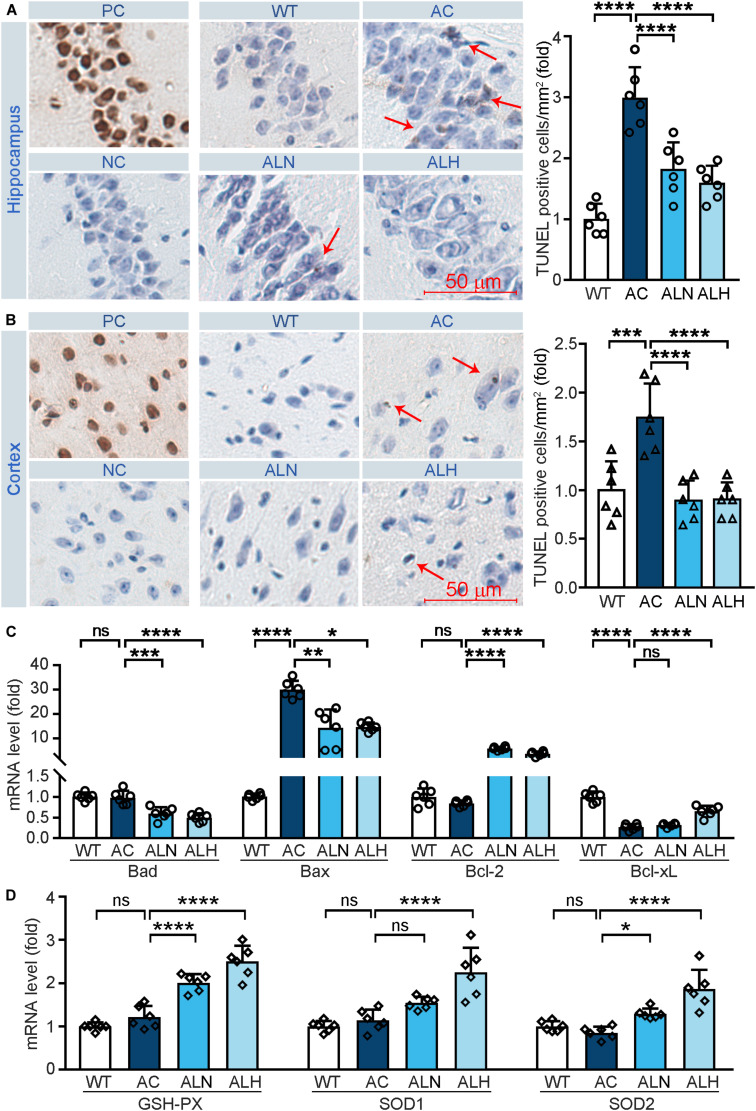
LongShengZhi Capsule Reduces Cell Death and Induces Antioxidant Enzymes in APP/PS1 Mouse Brain. At the end of the experiment as indicated in Figure 3A, mouse brain tissues were collected, followed by preparation of cross sections or total RNA. **(A,B)** the sections were used to conduct TUNEL staining to determine cell death (brown, arrow pointed) in THE CA1 region of the hippocampus **(A)** and cortex region **(B)**. PC, Positive control; NC, negative control (scale bar: 50 μm); **(C,D)** mRNA levels of apoptosis-related genes (Bad, Bax, Bcl-2, and Bcl-xL) and antioxidant genes (GSH-PX, SOD1, and SOD2) were detected by qRT-PCR. *p < 0.05; **p < 0.01; ***p < 0.001; ****p < 0.0001; ns, not significant (*n* = 6).

Free radical-induced oxidative stress usually occurs in the AD brain (Saito et al., 2019). Excessive oxidative stress promotes neuronal cell apoptosis/death and increases brain damage, which aggravates the AD process (Jiang et al., 2016). In addition, neurons in the CA1 region of hippocampus are more sensitive to age and pathological oxidative stress induced by cerebral ischemia in the AD process (Berkowitz et al., 2017). To determine the neuroprotective effect of LSZ *in vivo*, we performed TUNEL staining on brain tissues. Increased cell death in the CA1 region of the hippocampus (*F*_(__3_, _20__)_ = 28.97, *p* < 0.0001) (Figure 4A) and cortical areas (*F*_(__3_, _20__)_ = 13.93, *p* = 0.0003) (Figure 4B) in the AC group was observed, but LSZ treatment significantly reduced cell death in both regions by ∼39% and ∼46% in the hippocampus (*F*_(__3_, _20__)_ = 28.97, *p* < 0.0001) and ∼48% in the cortex (*F*_(__3_, _20__)_ = 13.93, *p* < 0.0001). Mechanistically, LSZ treatment significantly down-regulated mRNA expression of pro-apoptotic genes, Bax (AC vs. ALN, *F*_(__3_, _20__)_ = 68.19, *p* = 0.0011, AC vs. ALH, *F*_(__3_, _20__)_ = 68.19, *p* = 0.0324) and Bad (AC vs. ALN, *F*_(__3_, _20__)_ = 22.73, *p* = 0.0002; AC vs. ALH, *F*_(__3_, _20__)_ = 22.73, *p* < 0.0001), and activated mRNA expression of anti-apoptotic genes, Bcl-xL (AC vs, ALH, *F*_(__3_, _20__)_ = 65.88, *p* < 0.0001) and Bcl-2 (*F*_(__3_, _20__)_ = 135.2, *p* < 0.0001) in the brains of APP/PS1 mice (Figure 4C).

We also examined the mRNA levels of antioxidant genes to determine the association between the neuroprotection effect of LSZ and its antioxidative stress functions in the mouse brain. Compared with the WT group, mRNA levels of GSH-PX, SOD1, and SOD2 in the APP/PS1 mouse brain were slightly affected but potently increased by LSZ treatment (AC vs. ALH, AC vs. ALH, *F*_(__3_, _20__)_ = 46.99, *p* < 0.0001 for GSH-PX; AC vs. ALH, *F*_(__3_, _20__)_ = 24.16, *p* < 0.00015 for SOD1; AC vs. ALH, *F*_(__3_, _20__)_ = 19.22, *p* = 0.0194, AC vs. ALH, *F*_(__3_, _20__)_ = 19.22, *p* < 0.0001 for SOD2) (Figure 4D). These results show that LSZ can enhance mRNA expression of antioxidant factors and reduce cell death in the brains of APP/PS1 mice.

### LSZ Reduces β-Secretase and γ-Secretase Gene Expression and Inhibits Aβ Deposition

**FIGURE 5:**
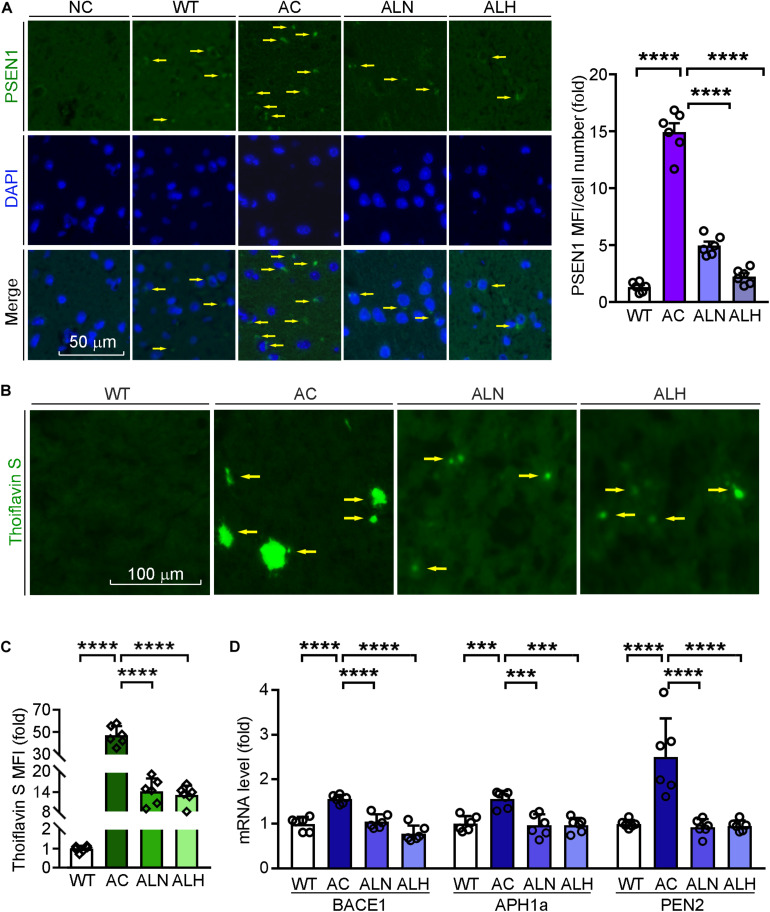
LongShengZhi Capsule Reduces PSEN1 Expression and Aβ Accumulation in APP/PS1 Mouse Brain. **(A)** PSEN1 protein expression (green) in mouse brain sections was determined by immunofluorescent staining with quantitation of MFI/cell number. The nucleus was stained with DAPI (blue). NC: Negative control (scale bar: 50 μm); **(B,C)** brain sections were performed thioflavin S staining with quantitation of Aβ plaques (scale bar: 100 μm); **(D)** mRNA expression of β-secretase and γ-secretase subunit (BACE1, APH1a, PEN2) was detected by qRT-PCR. ***p < 0.001, ****p < 0.0001 (*n* = 6).

Previous studies have shown that the overexpression of mutated PSEN1 in AD brains promotes β-secretase expression and influences γ-secretase activity to enhance Aβ secretion (De Strooper et al., 1998; Al-Atrache et al., 2019). To investigate the effect of LSZ on Aβ secretion and accumulation in APP/PS1 mice, we determined PSEN1 expression by immunofluorescent staining and Aβ accumulation by thioflavin S staining of mouse brain sections. We found in the AC group PSEN1 expression was increased to ∼0-fold of the WT group (*F*_(__3_, _20__)_ = 188.7, *p* < 0.0001), while the deposition of Aβ plaques was increased to ∼40-fold of the WT group (*F*_(__3_, _20__)_ = 192, *p* < 0.0001) (Figures 5A-C). However, PSEN1 expression (*F*_(__3_, _20__)_ = 188.7, *p* < 0.0001) and Aβ plaque deposition (*F*_(__3_, _20__)_ = 192, *p* < 0.0001) in brain tissues were significantly reduced by LSZ treatment.

Meanwhile, we indicated the specificity of thioflavin S to Aβ plaques by immunofluorescent staining with APP antibody, which can identify the β-C-terminal of APP (Supplementary Figure 2). Then we detected that LSZ also reduced the upregulated mRNA levels of BACE1 and γ-secretase subunits, APH1a and PEN2, compared to the AC group (*F*_(__3_, _20__)_ = 27.62, *p* < 0.0001 for BACE1; *F*_(__3_, _20__)_ = 13.08, *p* = 0.001 for APH1a; *F*_(__3_, _20__)_ = 46.12, *p* < 0.0001 for PEN2) (Figure 5D). These results indicate that LSZ can reduce Aβ deposition by inhibiting amyloidogenic pathway during the AD-like pathology in APP/PS1 mice.

### LSZ Reduces Inflammation and Hyperphosphorylation of Tau in AD Model Mice

**FIGURE 6:**
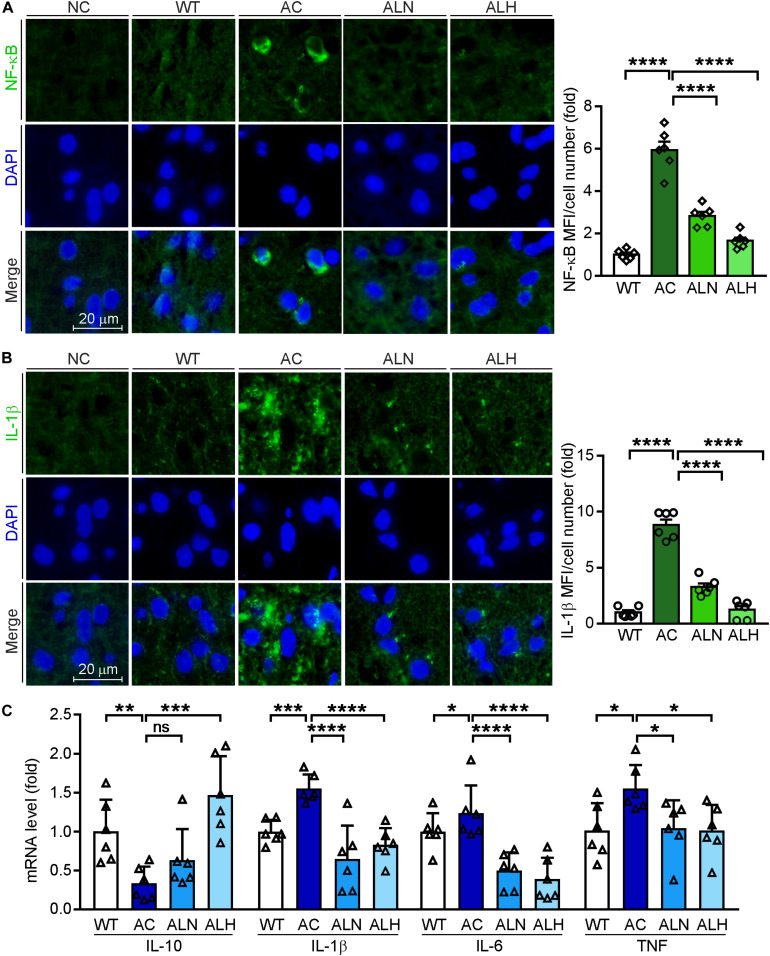
LongShengZhi Capsule Inhibits NF-κB, IL-1β, IL-6, and TNF, but Induces IL-10 Expression. Expression of NF-κB **(A)** and IL-1β **(B)** protein (green) in mouse brain sections was determined by immunofluorescent staining with quantitation of MFI/cell number. The nucleus was stained with DAPI (blue). NC, Negative control (scale bar: 20 μm); **(C)** mRNA levels of IL-10, IL-1β, IL-6, and TNF in the mouse brain was detected by qRT-PCR. *p < 0.05, **p < 0.01, ***p < 0.001, ****p < 0.0001; ns, not significant (*n* = 6).

**FIGURE 7:**
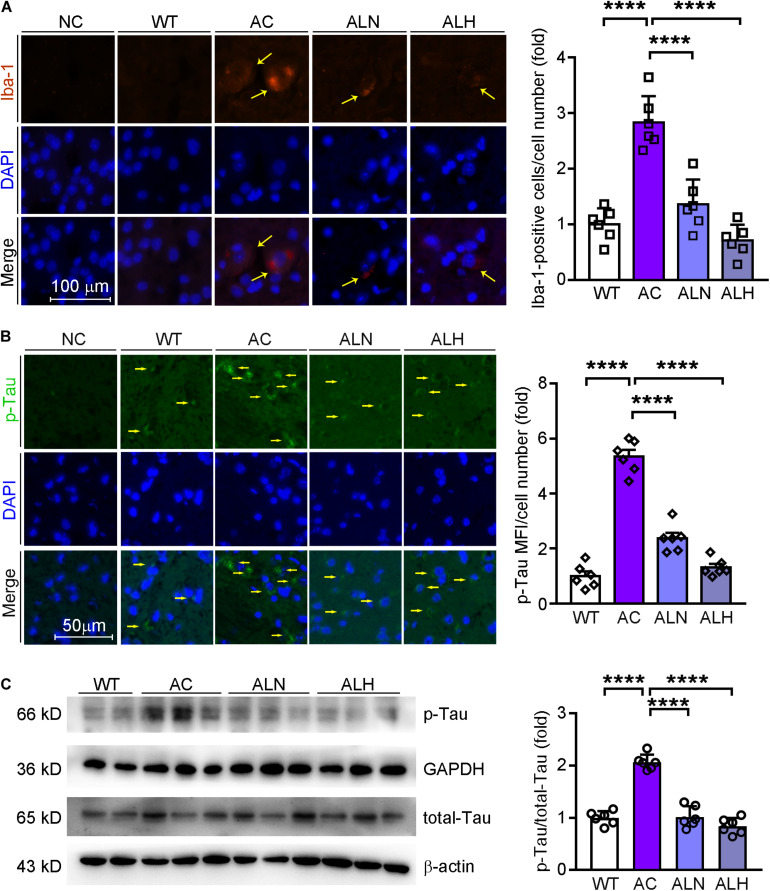
LongShengZhi Capsule Inhibits Microglia Activation and Tau Phosphorylation in APP/PS1 Mouse Brain. Iba-1 (red, **A**) in mouse brain sections was determined by immunofluorescent staining with quantitation of Iba-1-positive cells/cell number, and phosphorylated Tau (p-Tau, green, **B**) in mouse brain sections was determined by immunofluorescent staining with quantitation of MFI/cell number. The nucleus was stained with DAPI (blue); NC indicates the negative control (scale bar: 100 μm for A; 50 μm for B); **(C)** p-Tau and total Tau protein expression were determined by Western blot. Based on the band intensity, the ratio of p-Tau to total Tau was calculated. ****p < 0.0001 (*n* = 6).

Brain inflammation is common in AD patients. It is due to NF-κB-mediated production of inflammatory cytokines by activated microglia, such as IL-1β and TNF (Snow and Albensi, 2016). IL-1β can further promote Tau phosphorylation while TNF increases the β-cleavage of APP, thereby enhancing Aβ production/accumulation and the neuronal cell death (Ghosh et al., 2013). By performing immunofluorescent staining, we found that NF-κB (*F*_(__3_, _20__)_ = 82.88, *p* < 0.0001), IL-1β (*F*_(__3_, _20__)_ = 111.4, *p* < 0.0001) and Iba-1 (a marker for microglia activation, *F*_(__3_, _20__)_ = 31.01, *p* < 0.0001) were increased in the AC group compared with WT mice, but the increases were markedly inhibited by LSZ treatment (*F*_(__3_, _20__)_ = 82.88, *p* < 0.0001 for NF-κB; *F*_(__3_, _20__)_ = 111.4, *p* < 0.0001 for IL-1β; *F*_(__3_, _20__)_ = 31.01, *p* < 0.0001 for Iba-1) (Figures 6A,B, 7A). Similarly, compared with the AC group, LSZ in high dose significantly increased mRNA levels of anti-inflammatory cytokine, IL-10 (*F*_(__3_, _20__)_ = 12.4, *p* = 0.0009), and both doses of LSZ treatment decreased mRNA levels of pro-inflammatory cytokines, IL-1β (*F*_(__3_, _20__)_ = 26.63, *p* < 0.0001), IL-6 (*F*_(__3_, _20__)_ = 24.38, *p* < 0.0001), and TNF (AC vs, ALN, *F*_(__3_, _20__)_ = 3.799, *p* = 0.0459; AC vs, ALH, *F*_(__3_, _20__)_ = 3.799, *p* = 0.0323) (Figure 6C). We further examined Tau expression and its phosphorylation. Compared with the WT group, Tau phosphorylation in the AC group was elevated (*F*_(__3_, _20__)_ = 106.2, *p* < 0.0001 for p-Tau MFI/cell number; *F*_(__3_, _20__)_ = 72.59, *p* < 0.00013 for p-Tau/total Tau), but the elevation was significantly blocked by LSZ treatment (*F*_(__3_, _20__)_ = 106.2, *p* < 0.0001 for p-Tau MFI/cell number; *F*_(__3_, _20__)_ = 72.59, *p* < 0.0001 for p-Tau/total Tau), although neither the AD model nor LSZ treatment had an effect on Tau expression (Figure 7). Taken together, these results demonstrate that LSZ can reduce NF-κB-mediated brain inflammation and hyperphosphorylation of Tau in APP/PS1 mice.

## Discussion

In this study, we used APP/PS1 mice to investigate the therapeutic effect of LSZ on AD-like pathology. We clearly show that LSZ can improve the damaged cognitive functions in APP/PS1 mice (Figure 3). LSZ inhibited cell death in the brains of APP/PS1 mice and reduced oxidative stress and consequently regulated Bcl-2 family gene expression (Figure 4). LSZ also significantly inhibited glia activation and protein or mRNA expression of inflammatory cytokines (Figures 6, 7). Pathologically, LSZ reduced Aβ accumulation and Tau hyperphosphorylation (Figures 5, 7). Similar to the *in vivo* study, *in vitro* LSZ decreased L-Glu-induced ROS production and enhanced survival of HT-22 cells (Figure 1). The neuroprotective effect of LSZ was partly through the FAS/Bcl-2/p53 pathway and NF-κB-mediated inflammation response (Figure 2 and Supplementary Figure 1).

LongShengZhi Capsule as a traditional Chinese medicine for treatment of atherosclerotic cerebral infarction is usually used to treat hemiplegia and stroke caused by atherosclerosis with a high degree of safety. In our previous study, we showed LSZ has few side effects (Li et al., 2019; Ma et al., 2019; Xu et al., 2020). Similarly, in this study, we treated the animal with LSZ for 7 months and found no difference in bodyweight gain, food intake, and the external appearance between control and LSZ-treated groups, which further confirms the high degree of safety of the long-term LSZ treatment.

Oxidative stress and inflammation in the brain are considered important causes of AD. Many factors, such as brain injury, hypoxia, and insufficient cerebral blood flow, can cause excessive production of ROS in the brain. Recent studies have shown a significant decline in cerebral perfusion in the early stages of AD (Fouda et al., 2019). The early defect of cerebral perfusion reduces the removal of Aβ and exacerbates brain oxidative stress (Tarasoff-Conway et al., 2015). Oxidative stress impairs the normal growth of neurons and synapses, as well as the transmission of nerve signals, thereby promoting cognitive damage (Li et al., 2017). Therefore, enhancing cerebral perfusion could be a potential therapeutic for AD. Preclinical experiments have shown that LSZ can also increase cerebral blood flow in experimental animals and reduce cerebral vascular resistance. LSZ improves cerebral blood flow, which suggests an anti-AD potential by targeting Aβ clearing.

In terms of the composition, many components in LSZ have been proven to resist AD. For example, paeoniflorin, the most abundant component in LSZ, reduces the age-onset Aβ proteotoxicity by antioxidative stress (Ai et al., 2018). It has strong neuroprotective effects and cognitive preservation effects in intracerebroventricular streptozotocin-induced mice (Wang et al., 2018). Isoflavones, another high-content component in LSZ, have obvious neuroprotective effects (Wang et al., 2018). Isoflavones can attenuate oxidative stress and improve parameters related to AD-like pathology in D-galactose-treated C57BL/6J mice (Hsieh et al., 2009). These studies suggest that LSZ may protect against oxidative stress in AD through these active ingredients. We found LSZ significantly improved the damaged cognitive functions in 9-month-old APP/PS1 mice. Little has been reported thus far about the potential protective effects of LSZ on neurodegenerative diseases and its use as a prescribed medicine for treatment of cerebrovascular disease. However, many bioactive components in LSZ have been demonstrated to improve cognition. For example, paeoniflorin contained in LSZ can significantly improve the performance of aged wild-type rats in passive avoidance tasks (Baltina et al., 2019). Ferulic acid was reported to improve the escape latency of normal mice (Zafeer et al., 2019). These studies imply that LSZ may protect normal cognition against the damages caused by AD.

Furthermore, we determined that LSZ significantly reduced ROS levels in HT-22 cells and enhanced expression of antioxidant factors, SOD1, SOD2, and GSH-PX, in the APP/PS1 mouse brain. These results suggest that the anti-AD-like pathology effect of LSZ is partly associated with antioxidative stress. The oxidative stress–promoted cell death mainly occurs through mitochondrial pathways, including the Bcl-2 family, FAS, and p53. Aβ can induce expression of apoptosis-related factors by activating the FAS/FASL pathway (Morishima et al., 2001). Aβ can also promote p53 stabilization, which leads to neuronal damage (Lapresa et al., 2019). As expected, in addition to the decline in oxidative stress, cell death was significantly reduced by LSZ treatment both *in vivo* and *in vitro*. We detected that LSZ largely enhanced expression of anti-apoptotic factors Bcl-2 and Bcl-xL while inhibiting expression of pro-apoptotic factors Bax and Bad. We also determined L-Glu-increased expression of FAS and p53 in HT-22 cells was significantly inhibited by LSZ, while the regulation of FAS and the Bcl-2 family by LSZ was blocked by FAS siRNA, indicating LSZ enhances neuron survival partly through the FAS/Bcl-2/p53 pathway. We do not know from the pretreatment mode *in vitro* how long LSZ can be given to continue to exert neuroprotective effects after the onset of neuron toxic. Further exploration is required.

Interestingly, *in vivo*, we noticed higher levels of Bcl-2, SOD, and GSH-PX expression in the brains of APP/PS1 mice treated with LSZ than in wild-type mice. As an anti-apoptotic protein, although the promotion of Bcl-2 has led us to consider an increase in the risk of cancer, its overexpression has been proven to resist Aβ-induced PC12 cell death (Song et al., 2004). Moreover, acting as barriers for cells and body against free radicals, the changes in antioxidant factors SOD and GSH-PX in AD were controversial (Markesbery, 1997). In our study, we observed increased Bcl-2 and antioxidant enzymes levels in LSZ-treated AD mice, even higher than untreated wild type levels. Whether such activation for the long term may cause detrimental effect or not remains unclear and warrants further investigation. However, clinical and basic studies have not reported any adverse events. In the future, the dose titration study should be completed to identify the optimal dose range of LSZ for AD treatment, which can demonstrate its therapeutic effects while keeping antioxidative stress and cell death under control.

Meanwhile, we observed accumulation of Aβ plaques and hyperphosphorylation of Tau were significantly increased in APP/PS1 mouse brains, while LSZ at different doses obviously reduced these two biomarkers. The reduction of Aβ plaques is associated with the inhibition of the amyloidogenic pathway. PSEN1, a subunit of γ-secretase, not only catalyzes the final γ-cleavage of APP but also mediates oxidative stress–induced expression of β-secretase. Thus, activation of PSEN1 results in excessive Aβ production/accumulation in the AD process (Cai et al., 2018). Our results show that PSEN1 expression in AD mice was upregulated compared with the WT group, which was reversed by LSZ treatment. In addition, LSZ reduced transcription of BACE1 and another two subunits of γ-secretase, APH1a and PEN2, suggesting that LSZ can inhibit β-secretase and γ-secretase function/expression to decrease Aβ processing. However, we need to determine the exact mechanism by which LSZ affects normal or mutated PSEN1 expression since these transgenic mice express both, which can be recognized by the antibody used in this study.

Expression of p-Tau, another critical biomarker for AD development, can be enhanced by the high level of glia inflammation in the brain, particularly by NF-κB (Barron et al., 2017). In this study, we observed the microglia was significantly activated in the brains of APP/PS1 mice, while LSZ markedly reduced the activation. In addition, increased NF-κB and IL-1β in both APP/PS1 mouse brains and HT-22 cells were substantially reduced by LSZ treatment, suggesting the anti-inflammatory effects of LSZ also play an important role in its functions of anti-AD-like pathology.

In summary, here we report that the protection of LSZ against AD-like pathology may be related to its antioxidant and anti-inflammation functions. Although our study was completed with the APP/PS1 double transgenic mouse model, LSZ inhibits Aβ aggregation and Tau hyperphosphorylation, the basis for AD development. Therefore, we speculate that LSZ may also offer protection against AD in other kinds of AD models with similar rationales, such as 5 × FAD, 3 × Tg mice, and aluminum-induced AD models, all of which have high levels of Aβ plaques, NFTs, and oxidative stress in the brain.

Despite some interesting findings we obtained in this study, there are a few limitations that should be improved in future studies. These limitations include the following: (1) the effect of LSZ on some molecule should be further confirmed by determination of protein expression since mRNA expression does not always mean protein expression; (2) the exact mechanisms of LSZ on AD-like pathology need be further investigated; only its association with oxidative stress/cell death in the brain were reported in this study; (3) the neuroprotective effects of LSZ *in vitro* were only completed in the pretreatment regime; the therapeutic effect of LSZ after AD onset remains to be explored; and (4) we should investigate whether LSZ can also function well with the AD mouse model induced by cholinergic antagonist injection since a different rationale is involved in this kind of model, and such study can further broaden the potential application of LSZ in AD treatment.

## Conclusion

**FIGURE 8:**
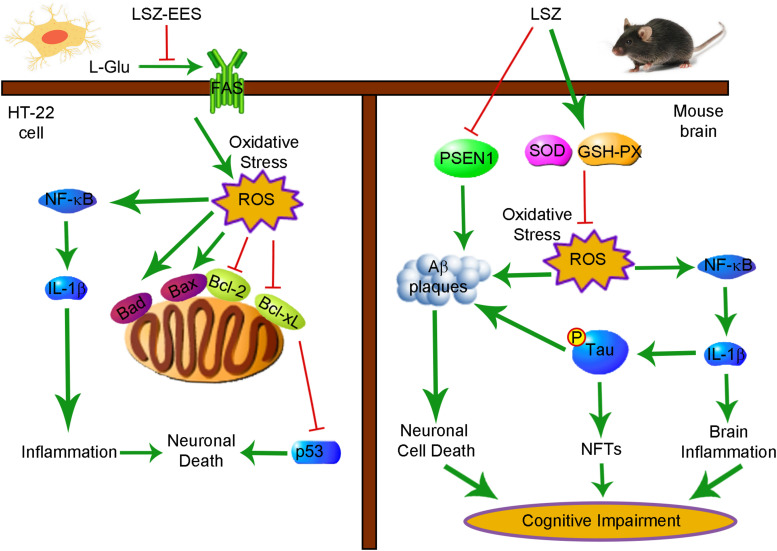
Schematic Showing the Neuroprotection and Anti-AD-Like Pathology Properties of LSZ on HT-22 Cells and APP/PS1 Mice Are Associated with Reducing Oxidative Stress and Inflammation. Bcl-2, B-cell lymphoma 2; Bcl-xL, B-cell lymphoma-extra large; Bad, Bcl-2 associated agonist of cell death; Bax, Bcl-2 associated X protein; FAS, Fas cell surface death receptor; GSH-PX, glutathione peroxidase; IL-1β, interleukin-1β; LSZ, LongShengZhi capsule; LSZ-EES, LSZ ethanol extract solution; L-Glu, L-glutamic acid; NF-κB, nuclear factor kappa-B; NFTs, neurofibrillary tangles; ROS, reactive oxygen species; SOD, superoxide dismutase.

We demonstrated that LSZ can significantly improve cognitive functions of APP/PS1 mice. LSZ enhances neuron survival in HT-22 cells partly by regulating the FAS/Bcl-2/p53 pathway. LSZ can also decrease oxidative stress and reduce inflammatory cytokine expression *in vitro*. Pathologically, LSZ inhibits AD-induced accumulation of Aβ plaques, hyperphosphorylation of Tau, cell death, and NF-κB-mediated inflammation, and promotes antioxidative gene expression in the brains of APP/PS1 mice (Figure 8). Based on the finding that APP/PS1 mice can spontaneously develop AD pathology, our study clearly demonstrates the therapeutic properties of LSZ on AD and suggests its potential clinical application.

## Data Availability Statement

The raw data supporting the conclusions of this article will be made available by the authors, without undue reservation.

## Ethics Statement

The animal study was reviewed and approved by the Institution Animal Ethics Committee of Hefei University of Technology.

## Author Contributions

JH and YD designed the study. ZY, XW, SHZ, PC, and YC performed the experiments. MY, CL, ZZ, and XY modified the file. SHZ and XY prepared the manuscript. All authors reviewed the results and approved the final version of the manuscript.

## Conflict of Interest

The authors declare that the research was conducted in the absence of any commercial or financial relationships that could be construed as a potential conflict of interest.
