# Supplementary material for: LongShengZhi Capsule Attenuates Alzheimer-Like Pathology in APP/PS1 Double Transgenic Mice by Reducing Neuronal Oxidative Stress and Inflammation
Source: Front Aging Neurosci. 2020 Nov 23;12:582455. doi: 10.3389/fnagi.2020.582455 (PMC7719723; doi:10.3389/fnagi.2020.582455)

Supplementary Material

# Supplementary Data

The data was conducted Shapiro-Wilk normality test and Brown-Forsythe test to evaluate if the values come from a Gaussian distribution. The mean ± SD for parametric data and exact p-values were listed in the table below.

**Shapiro-Wilk normality test to evaluate if the values come from a Gaussian distribution**

| **Figures** | | | **P value** | **Passed normality test (alpha = 0.05)?** |
| --- | --- | --- | --- | --- |
| **Figure 1A** | | | 0.1020 | Yes |
| **Figure 1C** | | | 0.2211 | Yes |
| **Figure 1D** | | | 0.0918 | Yes |
| **Figure 2A** | **Bad** | | 0.0731 | Yes |
|  | **Bax** | | 0.1592 | Yes |
|  | **Bcl-xL** | | 0.1233 | Yes |
|  | **FAS** | | 0.0736 | Yes |
| **Figure 2B** | **Bcl-2** | | 0.1932 | Yes |
|  | **Bad** | | 0.0591 | Yes |
|  | **p53** | | 0.4260 | Yes |
| **Figure 2C** | **Bax** | | 0.1788 | Yes |
|  | **NF-κB** | | 0.3097 | Yes |
|  | **IL-1β** | | 0.0538 | Yes |
| **Figure 3C** | | | 0.1548 | Yes |
| **Figure 3D** | | | 0.1901 | Yes |
| **Figure 3E** | | | 0.7971 | Yes |
| **Figure 3G** | | | 0.1339 | Yes |
| **Figure 3H** | | | 0.6528 | Yes |
| **Figure 3I** | | | 0.1076 | Yes |
| **Figure 4A** | | | 0.2045 | Yes |
| **Figure 4B** | | | 0.0530 | Yes |
| **Figure 4C** | **Bad** | | 0.3888 | Yes |
|  | **Bax** | | 0.1626 | Yes |
|  | **Bcl-2** | | 0.1891 | Yes |
|  | **Bcl-xL** | | 0.1227 | Yes |
| **Figure 4D** | **GSH-PX** | | 0.1478 | Yes |
|  | **SOD1** | | 0.0660 | Yes |
|  | **SOD2** | | 0.1129 | Yes |
| **Figure 5A** | | | 0.0806 | Yes |
| **Figure 5C** | | | 0.1574 | Yes |
| **Figure 5D** | **BACE1** | | 0.1888 | Yes |
|  | **APH1a** | | 0.1100 | Yes |
|  | **PEN2** | | 0.0732 | Yes |
| **Figure 6A** | | | 0.1288 | Yes |
| **Figure 6B** | | | 0.1089 | Yes |
| **Figure 6C** | **IL-10** | | 0.1273 | Yes |
|  | **IL-1β** | | 0.7001 | Yes |
|  | **IL-6** | | 0.2970 | Yes |
|  | **TNF** | | 0.7535 | Yes |
| **Figure 7A** | | | 0.1411 | Yes |
| **Figure 7B** | | | 0.1228 | Yes |
| **Figure 7C** | | | 0.1041 | Yes |
| **Figure S1A** | | **FAS** | 0.1286 | Yes |
|  |  | **Bcl-xL** | 0.1121 | Yes |
|  |  | **Bad** | 0.1040 | Yes |
|  |  | **Bax** | 0.0647 | Yes |
| **Figure S1C** | | **FAS** | 0.1054 | Yes |
|  |  | **Bax** | 0.1128 | Yes |
|  |  | **Bcl-2** | 0.1023 | Yes |

**White test to evaluate the variance heterogeneity for the data**

| **Figures** | | | **P value** | **Do the data have variance heterogeneity？** |
| --- | --- | --- | --- | --- |
| **Figure 1A** | | | 0.097 | No |
| **Figure 1C** | | | 0.416 | No |
| **Figure 1D** | | | 0.231 | No |
| **Figure 2A** | **Bad** | | 0.304 | No |
|  | **Bax** | | 0.141 | No |
|  | **Bcl-xL** | | 0.061 | No |
|  | **FAS** | | 0.085 | No |
| **Figure 2B** | **Bcl-2** | | 0.371 | No |
|  | **Bad** | | 0.129 | No |
|  | **p53** | | 0.431 | No |
| **Figure 2C** | **Bax** | | 0.408 | No |
|  | **NF-κB** | | 0.15 | No |
|  | **IL-1β** | | 0.152 | No |
| **Figure 3C** | | | 0.056 | No |
| **Figure 3D** | | | 0.443 | No |
| **Figure 3E** | | | 0.726 | No |
| **Figure 3G** | | | 0.077 | No |
| **Figure 3H** | | | 0.244 | No |
| **Figure 3I** | | | 0.34 | No |
| **Figure 4A** | | | 0.230 | No |
| **Figure 4B** | | | 0.086 | No |
| **Figure 4C** | **Bad** | | 0.396 | No |
|  | **Bax** | | 0.084 | No |
|  | **Bcl-2** | | 0.150 | No |
|  | **Bcl-xL** | | 0.167 | No |
| **Figure 4D** | **GSH-PX** | | 0.102 | No |
|  | **SOD1** | | 0.16 | No |
|  | **SOD2** | | 0.095 | No |
| **Figure 5A** | | | 0.084 | No |
| **Figure 5C** | | | 0.16 | No |
| **Figure 5D** | **BACE1** | | 0.471 | No |
|  | **APH1a** | | 0.281 | No |
|  | **PEN2** | | 0.111 | No |
| **Figure 6A** | | | 0.076 | No |
| **Figure 6B** | | | 0.103 | No |
| **Figure 6C** | **IL-10** | | 0.136 | No |
|  | **IL-1β** | | 0.353 | No |
|  | **IL-6** | | 0.639 | No |
|  | **TNF** | | 0.950 | No |
| **Figure 7A** | | | 0.158 | No |
| **Figure 7B** | | | 0.397 | No |
| **Figure 7C** | | | 0.432 | No |
| **Figure S1A** | | **FAS** | 0.08 | No |
|  |  | **Bcl-xL** | 0.318 | No |
|  |  | **Bad** | 0.77 | No |
|  |  | **Bax** | 0.295 | No |
| **Figure S1C** | | **FAS** | 0.055 | No |
|  |  | **Bax** | 0.056 | No |
|  |  | **Bcl-2** | 0.25 | No |

**Brown-Forsythe test to evaluate the variance homogeneity for the data**

| **Figures** | | | **P value** | **Are SDs significantly different (P < 0.05)?** |
| --- | --- | --- | --- | --- |
| **Figure 1A** | | | 0.0763 | No |
| **Figure 1C** | | | 0.8178 | No |
| **Figure 1D** | | | 0.7665 | No |
| **Figure 2A** | **Bad** | | 0.5447 | No |
|  | **Bax** | | 0.2795 | No |
|  | **Bcl-xL** | | 0.3737 | No |
|  | **FAS** | | 0.1100 | No |
| **Figure 2B** | **Bcl-2** | | 0.6167 | No |
|  | **Bad** | | 0.2678 | No |
|  | **p53** | | 0.7834 | No |
| **Figure 2C** | **Bax** | | 0.4852 | No |
|  | **NF-κB** | | 0.1478 | No |
|  | **IL-1β** | | 0.2029 | No |
| **Figure 3C** | | | 0.1352 | No |
| **Figure 3D** | | | 0.7363 | No |
| **Figure 3E** | | | 0.7481 | No |
| **Figure 3G** | | | 0.4481 | No |
| **Figure 3H** | | | 0.3213 | No |
| **Figure 3I** | | | 0.1987 | No |
| **Figure 4A** | | | 0.7054 | No |
| **Figure 4B** | | | 0.2204 | No |
| **Figure 4C** | **Bad** | | 0.5667 | No |
|  | **Bax** | | 0.0527 | No |
|  | **Bcl-2** | | 0.6899 | No |
|  | **Bcl-xL** | | 0.1962 | No |
| **Figure 4D** | **GSH-PX** | | 0.1165 | No |
|  | **SOD1** | | 0.0662 | No |
|  | **SOD2** | | 0.0778 | No |
| **Figure 5A** | | | 0.0705 | No |
| **Figure 5C** | | | 0.0824 | No |
| **Figure 5D** | **BACE1** | | 0.8793 | No |
|  | **APH1a** | | 0.5950 | No |
|  | **PEN2** | | 0.1247 | No |
| **Figure 6A** | | | 0.1243 | No |
| **Figure 6B** | | | 0.0605 | No |
| **Figure 6C** | **IL-10** | | 0.1232 | No |
|  | **IL-1β** | | 0.5420 | No |
|  | **IL-6** | | 0.7808 | No |
|  | **TNF** | | 0.9512 | No |
| **Figure 7A** | | | 0.6971 | No |
| **Figure 7B** | | | 0.4419 | No |
| **Figure 7C** | | | 0.7521 | No |
| **Figure S1A** | | **FAS** | 0.1180 | No |
|  |  | **Bcl-xL** | 0.5711 | No |
|  |  | **Bad** | 0.1484 | No |
|  |  | **Bax** | 0.3103 | No |
| **Figure S1C** | | **FAS** | 0.1737 | No |
|  |  | **Bax** | 0.6608 | No |
|  |  | **Bcl-2** | 0.2405 | No |

**The mean ± SD for parametric data**

**Figure 1A**

| **Group** | **LSZ-EES (μg/mL)** | **0** | **0** | **10** | **10** | **20** | **20** |
| --- | --- | --- | --- | --- | --- | --- | --- |
|  | **L-Glu (mM)** | **0** | **20** | **0** | **20** | **0** | **20** |
| Mean ± SD | | 1 ± 0.02843 | 1.979 ± 0.3512 | 0.9424 ± 0.0984 | 1.227 ± 0.0810 | 0.8821 ± 0.0842 | 1.329 ± 0.0988 |

**Figure 1C**

| **Group (LSZ-EES μg/mL)** | **0** | **5** | **10** | **20** | **30** |
| --- | --- | --- | --- | --- | --- |
| Mean ± SD | 1 ± 0.1686 | 1.098 ± 0.2368 | 0.9492 ± 0.1268 | 0.9898 ± 0.1768 | 1.125 ± 0.2378 |

**Figure 1D**

| **Group** | **LSZ-EES (μg/mL)** | **0** | **0** | **10** | **20** |
| --- | --- | --- | --- | --- | --- |
|  | **L-Glu (mM)** | **0** | **20** | **20** | **20** |
| Mean ± SD | | 0.9984 ± 0.07218 | 0.6047 ± 0.07448 | 0.8605 ± 0.06784 | 0.9074 ± 0.04114 |

**Figure 2A** Bad mRNA level

| **Group** | **LSZ-EES (μg/mL)** | **0** | **0** | **10** | **10** |
| --- | --- | --- | --- | --- | --- |
|  | **L-Glu (mM)** | **0** | **20** | **0** | **20** |
| Mean ± SD | | 1 ± 0.2754 | 5.034 ± 0.4107 | 0.508 ± 0.2168 | 0.904 ± 0.2454 |

**Figure 2A** Bax mRNA level

| **Group** | **LSZ-EES (μg/mL)** | **0** | **0** | **10** | **10** |
| --- | --- | --- | --- | --- | --- |
|  | **L-Glu (mM)** | **0** | **20** | **0** | **20** |
| Mean ± SD | | 1.01 ± 0.1628 | 2.076 ± 0.5562 | 0.784 ± 0.06986 | 1.242 ± 0.1907 |

**Figure 2A** Bcl-xL mRNA level

| **Group** | **LSZ-EES (μg/mL)** | **0** | **0** | **10** | **10** |
| --- | --- | --- | --- | --- | --- |
|  | **L-Glu (mM)** | **0** | **20** | **0** | **20** |
| Mean ± SD | | 1.01 ± 0.1821 | 0.468 ± 0.1043 | 1.864 ± 0.3505 | 2.746 ± 0.8521 |

**Figure 2A** FAS mRNA level

| **Group** | **LSZ-EES (μg/mL)** | **0** | **0** | **10** | **10** |
| --- | --- | --- | --- | --- | --- |
|  | **L-Glu (mM)** | **0** | **20** | **0** | **20** |
| Mean ± SD | | 1.004 ± 0.1141 | 23.67 ± 3.122 | 1.738 ± 0.2597 | 4.958 ± 0.5501 |

**Figure 2B** Bcl-2 protein expression

| **Group** | **LSZ-EES (μg/mL)** | **0** | **0** | **10** | **20** |
| --- | --- | --- | --- | --- | --- |
|  | **L-Glu (mM)** | **0** | **20** | **20** | **20** |
| Mean ± SD | | 0.9999 ± 0.255 | 0.7458 ± 0.1241 | 1.438 ± 0.2062 | 1.317 ± 0.0998 |

**Figure 2B** Bad protein expression

| **Group** | **LSZ-EES (μg/mL)** | **0** | **0** | **10** | **20** |
| --- | --- | --- | --- | --- | --- |
|  | **L-Glu (mM)** | **0** | **20** | **20** | **20** |
| Mean ± SD | | 1 ± 0.1732 | 1.071 ± 0.1004 | 0.6282 ± 0.03353 | 0.6711 ± 0.07473 |

**Figure 2B** p53 protein expression

| **Group** | **LSZ-EES (μg/mL)** | **0** | **0** | **10** | **20** |
| --- | --- | --- | --- | --- | --- |
|  | **L-Glu (mM)** | **0** | **20** | **20** | **20** |
| Mean ± SD | | 1 ± 0.07265 | 1.302 ± 0.1022 | 0.8695 ± 0.1004 | 0.6493 ± 0.05665 |

**Figure 2C** Bax protein expression

| **Group** | **LSZ-EES (μg/mL)** | **0** | **0** | **10** | **20** |
| --- | --- | --- | --- | --- | --- |
|  | **L-Glu (mM)** | **0** | **20** | **20** | **20** |
| Mean ± SD | | 1 ± 0.1629 | 1.301 ± 0.1964 | 0.9226 ± 0.1096 | 1.08 ± 0.09394 |

**Figure 2C** NF-κB protein expression

| **Group** | **LSZ-EES (μg/mL)** | **0** | **0** | **10** | **20** |
| --- | --- | --- | --- | --- | --- |
|  | **L-Glu (mM)** | **0** | **20** | **20** | **20** |
| Mean ± SD | | 1 ± 0.2014 | 1.957 ± 0.192 | 1.026 ± 0.2827 | 1.025 ± 0.5335 |

**Figure 2C** IL-1β protein expression

| **Group** | **LSZ-EES (μg/mL)** | **0** | **0** | **10** | **20** |
| --- | --- | --- | --- | --- | --- |
|  | **L-Glu (mM)** | **0** | **20** | **20** | **20** |
| Mean ± SD | | 1.046 ± 0.0974 | 1.435 ± 0.04193 | 0.6897 ± 0.04161 | 0.6921 ± 0.04261 |

**Figure 3C** Escape latency

| **Group** | **WT** | **AC** | **ALN** | **ALH** |
| --- | --- | --- | --- | --- |
| Mean ± SD | 19 ± 4.014 | 57.14 ± 3.235 | 29.63 ± 5.423 | 26.55 ± 10.14 |

**Figure 3D** Platform crossings

| **Group** | **WT** | **AC** | **ALN** | **ALH** |
| --- | --- | --- | --- | --- |
| Mean ± SD | 3.833 ± 0.7528 | 1.167 ± 1.169 | 3 ± 0.8944 | 1.833 ± 0.7528 |

**Figure 3E** Times of passes through the target quadrant

| **Group** | **WT** | **AC** | **ALN** | **ALH** |
| --- | --- | --- | --- | --- |
| Mean ± SD | 9 ± 1.789 | 4.667 ± 1.211 | 8.5 ± 1.517 | 7.667 ± 1.862 |

**Figure 3G** New arm crossings

| **Group** | **WT** | **AC** | **ALN** | **ALH** |
| --- | --- | --- | --- | --- |
| Mean ± SD | 8.5 ± 1.378 | 3.333 ± 1.033 | 6.667 ± 2.338 | 4.333 ± 1.211 |

**Figure 3H** Distance in new arm

| **Group** | **WT** | **AC** | **ALN** | **ALH** |
| --- | --- | --- | --- | --- |
| Mean ± SD | 5.076 ± 1.009 | 2.604 ± 0.4821 | 4.219 ± 1.064 | 3.875 ± 0.6112 |

**Figure 3I** Time in new arm

| **Group** | **WT** | **AC** | **ALN** | **ALH** |
| --- | --- | --- | --- | --- |
| Mean ± SD | 100.5 ± 22.72 | 49.65 ± 7.589 | 74.05 ± 12.84 | 54.79 ± 7.858 |

**Figure 4A** TUNEL positive

| **Group** | **WT** | **AC** | **ALN** | **ALH** |
| --- | --- | --- | --- | --- |
| Mean ± SD | 1.007 ± 0.2468 | 2.997 ± 0.4961 | 1.824 ± 0.4371 | 1.598 ± 0.2818 |

**Figure 4B** TUNEL positive

| **Group** | **WT** | **AC** | **ALN** | **ALH** |
| --- | --- | --- | --- | --- |
| Mean ± SD | 1 ± 0.2964 | 1.742 ± 0.3534 | 0.8925 ± 0.2057 | 0.9032 ± 0.1779 |

**Figure 4C** Bad mRNA level

| **Group** | **WT** | **AC** | **ALN** | **ALH** |
| --- | --- | --- | --- | --- |
| Mean ± SD | 1.008 ± 0.09698 | 0.983 ± 0.1701 | 0.5999 ± 0.1511 | 0.4998 ± 0.1034 |

**Figure 4C** Bax mRNA level

| **Group** | **WT** | **AC** | **ALN** | **ALH** |
| --- | --- | --- | --- | --- |
| Mean ± SD | 1.011 ± 0.07436 | 25.59 ± 3.641 | 17.8 ± 3.236 | 20.48 ± 4.016 |

**Figure 4C** Bcl-2 mRNA level

| **Group** | **WT** | **AC** | **ALN** | **ALH** |
| --- | --- | --- | --- | --- |
| Mean ± SD | 1 ± 0.2051 | 0.9027 ± 0.1534 | 3.057 ± 0.2792 | 2.137 ± 0.2046 |

**Figure 4C** Bcl-xL mRNA level

| **Group** | **WT** | **AC** | **ALN** | **ALH** |
| --- | --- | --- | --- | --- |
| Mean ± SD | 1 ± 0.1307 | 0.2694 ± 0.07476 | 0.318 ± 0.04282 | 0.6528 ± 0.132 |

**Figure 4D** GSH-PX mRNA level

| **Group** | **WT** | **AC** | **ALN** | **ALH** |
| --- | --- | --- | --- | --- |
| Mean ± SD | 1 ± 0.09593 | 1.219 ± 0.2578 | 2.014 ± 0.1997 | 2.506 ± 0.3664 |

**Figure 4D** SOD1 mRNA level

| **Group** | **WT** | **AC** | **ALN** | **ALH** |
| --- | --- | --- | --- | --- |
| Mean ± SD | 1.004 ± 0.1242 | 1.144 ± 0.2482 | 1.501 ± 0.1059 | 2.091 ± 0.3808 |

**Figure 4D** SOD2 mRNA level

| **Group** | **WT** | **AC** | **ALN** | **ALH** |
| --- | --- | --- | --- | --- |
| Mean ± SD | 1.003 ± 0.1197 | 0.859 ± 0.1395 | 1.295 ± 0.1211 | 1.866 ± 0.4463 |

**Figure 5A** PSEN1 MFI/cell number

| **Group** | **WT** | **AC** | **ALN** | **ALH** |
| --- | --- | --- | --- | --- |
| Mean ± SD | 1 ± 0.3241 | 12.5 ± 1.138 | 3.731 ± 0.6435 | 1.699 ± 0.5065 |

**Figure 5C** Thioflavin S MFI

| **Group** | **WT** | **AC** | **ALN** | **ALH** |
| --- | --- | --- | --- | --- |
| Mean ± SD | 1.003 ± 0.1667 | 46.21 ± 3.047 | 14.3 ± 3.951 | 13.21 ± 2.82 |

**Figure 5D** BACE1 mRNA level

| **Group** | **WT** | **AC** | **ALN** | **ALH** |
| --- | --- | --- | --- | --- |
| Mean ± SD | 1 ± 0.1543 | 1.553 ± 0.0966 | 1.052 ± 0.1631 | 0.7821 ± 0.1804 |

**Figure 5D** APH1a mRNA level

| **Group** | **WT** | **AC** | **ALN** | **ALH** |
| --- | --- | --- | --- | --- |
| Mean ± SD | 1.005 ± 0.1693 | 1.553 ± 0.1863 | 0.9659 ± 0.2474 | 0.9709 ± 0.1619 |

**Figure 5D** PEN2 mRNA level

| **Group** | **WT** | **AC** | **ALN** | **ALH** |
| --- | --- | --- | --- | --- |
| Mean ± SD | 1 ± 0.0902 | 2.838 ± 0.6351 | 0.9222 ± 0.1854 | 0.9545 ± 0.1191 |

**Figure 6A** NF-κB MFI/cell number

| **Group** | **WT** | **AC** | **ALN** | **ALH** |
| --- | --- | --- | --- | --- |
| Mean ± SD | 1 ± 0.2363 | 5.941 ± 0.9871 | 2.827 ± 0.4776 | 1.657 ± 0.3632 |

**Figure 6B** IL-1β MFI/cell number

| **Group** | **WT** | **AC** | **ALN** | **ALH** |
| --- | --- | --- | --- | --- |
| Mean ± SD | 1 ± 0.4638 | 8.811 ± 1.208 | 3.272 ± 0.7572 | 1.25 ± 0.7683 |

**Figure 6C** IL-10 mRNA level

| **Group** | **WT** | **AC** | **ALN** | **ALH** |
| --- | --- | --- | --- | --- |
| Mean ± SD | 1.004 ± 0.4055 | 0.3852 ± 0.1819 | 0.4656 ± 0.1104 | 1.303 ± 0.4037 |

**Figure 6C** IL-1β mRNA level

| **Group** | **WT** | **AC** | **ALN** | **ALH** |
| --- | --- | --- | --- | --- |
| Mean ± SD | 1 ± 0.151 | 1.553 ± 0.1795 | 0.5316 ± 0.2544 | 0.8295 ± 0.215 |

**Figure 6C** IL-6 mRNA level

| **Group** | **WT** | **AC** | **ALN** | **ALH** |
| --- | --- | --- | --- | --- |
| Mean ± SD | 1 ± 0.2361 | 1.401 ± 0.1779 | 0.5012 ± 0.2311 | 0.3885 ± 0.274 |

**Figure 6C** TNF mRNA level

| **Group** | **WT** | **AC** | **ALN** | **ALH** |
| --- | --- | --- | --- | --- |
| Mean ± SD | 1.011 ± 0.3532 | 1.556 ± 0.2978 | 1.042 ± 0.3603 | 1.011 ± 0.3316 |

**Figure 7A** Iba-1-positive cells/cell number

| **Group** | **WT** | **AC** | **ALN** | **ALH** |
| --- | --- | --- | --- | --- |
| Mean ± SD | 1 ± 0.2887 | 2.832± 0.4766 | 1.358 ± 0.4492 | 0.7088 ± 0.2857 |

**Figure 7B** p-Tau MFI/cell number

| **Group** | **WT** | **AC** | **ALN** | **ALH** |
| --- | --- | --- | --- | --- |
| Mean ± SD | 1 ± 0.4233 | 5.34 ± 0.601 | 2.373 ± 0.493 | 1.312 ± 0.3138 |

**Figure 7C** p-Tau/total-Tau

| **Group** | **WT** | **AC** | **ALN** | **ALH** |
| --- | --- | --- | --- | --- |
| Mean ± SD | 1 ± 0.129 | 2.065 ± 0.1476 | 1.02 ± 0.2039 | 0.8441 ± 0.155 |

**Supplementary Figure 1A** FAS mRNA level

| **Group** | **LSZ-EES (μg/mL)** | **siCtrl** | | | | **siFAS** | | | |
| --- | --- | --- | --- | --- | --- | --- | --- | --- | --- |
|  |  | **0** | **0** | **10** | **20** | **0** | **0** | **10** | **20** |
|  | **L-Glu (mM)** | **0** | **20** | **0** | **20** | **0** | **20** | **0** | **20** |
| Mean ± SD | | 1 ± 0.1661 | 0.268 ± 0.0342 | 2.382 ± 0.3337 | 0.622 ± 0.1316 | 1.704 ± 0.1791 | 0.472 ± 0.111 | 0.978 ± 0.1481 | 0.284 ± 0.1161 |

**Supplementary Figure 1A** Bcl-xL mRNA level

| **Group** | **LSZ-EES (μg/mL)** | **siCtrl** | | | | **siFAS** | | | |
| --- | --- | --- | --- | --- | --- | --- | --- | --- | --- |
|  |  | **0** | **0** | **10** | **20** | **0** | **0** | **10** | **20** |
|  | **L-Glu (mM)** | **0** | **20** | **0** | **20** | **0** | **20** | **0** | **20** |
| Mean ± SD | | 1.006 ± 0.1075 | 1.27 ± 0.1396 | 0.359 ± 0.0204 | 0.414 ± 0.0713 | 0.703 ± 0.0533 | 0.493 ± 0.0597 | 0.918 ± 0.1017 | 0.483 ± 0.1143 |

**Supplementary Figure 1A** Bad mRNA level

| **Group** | **LSZ-EES (μg/mL)** | **siCtrl** | | | | **siFAS** | | | |
| --- | --- | --- | --- | --- | --- | --- | --- | --- | --- |
|  |  | **0** | **0** | **10** | **20** | **0** | **0** | **10** | **20** |
|  | **L-Glu (mM)** | **0** | **20** | **0** | **20** | **0** | **20** | **0** | **20** |
| Mean ± SD | | 0.999 ± 0.1572 | 0.415 ± 0.0487 | 2.108 ± 0.3144 | 0.868 ± 0.0680 | 1.518 ± 0.1388 | 0.784 ± 0.2463 | 1.114 ± 0.1882 | 0.590 ± 0.0956 |

**Supplementary Figure 1A** Bax mRNA level

| **Group** | **LSZ-EES (μg/mL)** | **siCtrl** | | | | **siFAS** | | | |
| --- | --- | --- | --- | --- | --- | --- | --- | --- | --- |
|  |  | **0** | **0** | **10** | **20** | **0** | **0** | **10** | **20** |
|  | **L-Glu (mM)** | **0** | **20** | **0** | **20** | **0** | **20** | **0** | **20** |
| Mean ± SD | | 1.016 ± 0.0659 | 0.479 ± 0.0831 | 1.619 ± 0.124 | 0.624 ± 0.0340 | 0.821 ± 0.1493 | 0.481 ± 0.1019 | 0.978 ± 0.0876 | 0.463 ± 0.0465 |

**Supplementary Figure 1C** FAS protein expression

| **Group** | **LSZ-EES (μg/mL)** | **siCtrl** | | | | **siFAS** | | | |
| --- | --- | --- | --- | --- | --- | --- | --- | --- | --- |
|  |  | **0** | **0** | **10** | **20** | **0** | **0** | **10** | **20** |
|  | **L-Glu (mM)** | **0** | **20** | **0** | **20** | **0** | **20** | **0** | **20** |
| Mean ± SD | | 1 ± 0.0676 | 1.245 ± 0.0808 | 0.513 ± 0.0348 | 0.514 ± 0.0294 | 0.558 ± 0.0237 | 0.492 ± 0.0236 | 0.428 ± 0.0179 | 0.496 ± 0.0446 |

**Supplementary Figure 1C** Bax protein expression

| **Group** | **LSZ-EES (μg/mL)** | **siCtrl** | | | | **siFAS** | | | |
| --- | --- | --- | --- | --- | --- | --- | --- | --- | --- |
|  |  | **0** | **0** | **10** | **20** | **0** | **0** | **10** | **20** |
|  | **L-Glu (mM)** | **0** | **20** | **0** | **20** | **0** | **20** | **0** | **20** |
| Mean ± SD | | 1 ± 0.1312 | 1.165 ± 0.0513 | 0.364 ± 0.047 | 0.421 ± 0.0756 | 0.363 ± 0.0285 | 0.349 ± 0.0315 | 0.324 ± 0.0161 | 0.253 ± 0.0219 |

**Supplementary Figure 1C** Bcl-2 protein expression

| **Group** | **LSZ-EES (μg/mL)** | **siCtrl** | | | | **siFAS** | | | |
| --- | --- | --- | --- | --- | --- | --- | --- | --- | --- |
|  |  | **0** | **0** | **10** | **20** | **0** | **0** | **10** | **20** |
|  | **L-Glu (mM)** | **0** | **20** | **0** | **20** | **0** | **20** | **0** | **20** |
| Mean ± SD | | 1 ± 0.0701 | 0.551 ± 0.0462 | 0.934 ± 0.0394 | 0.907 ± 0.0181 | 0.558 ± 0.0533 | 0.623 ± 0.0237 | 0.672 ± 0.0346 | 0.616 ± 0.0193 |

**Exact p-values**

**Figure 1A**

| **Two-way anova test** | **Significant?** | **Summary** | **Adjusted P Value** |
| --- | --- | --- | --- |
| LSZ (0 μg/mL) + L-Glu (0 mM)  vs. LSZ (0 μg/mL) + L-Glu (20 mM) | Yes | *** | 0.0002 |
| LSZ (0 μg/mL) + L-Glu (20 mM)  vs. LSZ (10 μg/mL) + L-Glu (20 mM) | Yes | ** | 0.0015 |
| LSZ (0 μg/mL) + L-Glu (20 mM)  vs. LSZ (20 μg/mL) + L-Glu (20 mM) | Yes | ** | 0.0017 |

**Figure 1C**

| **One-way anova test** | **Significant?** | **Summary** | **Adjusted P Value** |
| --- | --- | --- | --- |
| LSZ (0 μg/mL) vs. LSZ (5 μg/mL) | No | ns | 0.8964 |
| LSZ (0 μg/mL) vs. LSZ (10 μg/mL) | No | ns | 0.9899 |
| LSZ (0 μg/mL) vs. LSZ (20 μg/mL) | No | ns | 0.9999 |
| LSZ (0 μg/mL) vs. LSZ (30 μg/mL) | No | ns | 0.7851 |

**Figure 1D**

| **One-way anova test** | **Significant?** | **Summary** | **Adjusted P Value** |
| --- | --- | --- | --- |
| LSZ (0 μg/mL) + L-Glu (0 mM) vs. LSZ (0 μg/mL) + L-Glu (20 mM) | Yes | *** | 0.0003 |
| LSZ (0 μg/mL) + L-Glu (20 mM) vs. LSZ (10 μg/mL) + L-Glu (20 mM) | Yes | *** | 0.0009 |
| LSZ (0 μg/mL) + L-Glu (20 mM) vs. LSZ (20 μg/mL) + L-Glu (20 mM) | Yes | *** | 0.0007 |

**Figure 2A** Bad mRNA level

| **Two-way anova test** | **Significant?** | **Summary** | **Adjusted P Value** |
| --- | --- | --- | --- |
| LSZ (0 μg/mL) + L-Glu (0 mM) vs. LSZ (0 μg/mL) + L-Glu (20 mM) | Yes | **** | <0.0001 |
| LSZ (0 μg/mL) + L-Glu (20 mM) vs. LSZ (10 μg/mL) + L-Glu (20 mM) | Yes | **** | <0.0001 |
| LSZ (0 μg/mL) vs. LSZ (10 μg/mL) | No | ns | 0.1057 |
| LSZ (10 μg/mL) vs. LSZ (10 μg/mL) + L-Glu (20 mM) | No | ns | 0.2687 |

**Figure 2A** Bax mRNA level

| **Two-way anova test** | **Significant?** | **Summary** | **Adjusted P Value** |
| --- | --- | --- | --- |
| LSZ (0 μg/mL) + L-Glu (0 mM) vs. LSZ (0 μg/mL) + L-Glu (20 mM) | Yes | *** | 0.0003 |
| LSZ (0 μg/mL) + L-Glu (20 mM) vs. LSZ (10 μg/mL) + L-Glu (20 mM) | Yes | ** | 0.0033 |
| LSZ (0 μg/mL) vs. LSZ (10 μg/mL) | No | ns | 0.8379 |
| LSZ (10 μg/mL) vs. LSZ (10 μg/mL) + L-Glu (20 mM) | No | ns | 0.1742 |

**Figure 2A** Bcl-xL mRNA level

| **Two-way anova test** | **Significant?** | **Summary** | **Adjusted P Value** |
| --- | --- | --- | --- |
| LSZ (0 μg/mL) + L-Glu (0 mM) vs. LSZ (0 μg/mL) + L-Glu (20 mM) | Yes | * | 0.0403 |
| LSZ (0 μg/mL) + L-Glu (20 mM) vs. LSZ (10 μg/mL) + L-Glu (20 mM) | Yes | **** | <0.0001 |
| LSZ (0 μg/mL) vs. LSZ (10 μg/mL) | Yes | ** | 0.0019 |
| LSZ (10 μg/mL) vs. LSZ (10 μg/mL) + L-Glu (20 mM) | Yes | ** | 0.0014 |

**Figure 2A** FAS mRNA level

| **Two-way anova test** | **Significant?** | **Summary** | **Adjusted P Value** |
| --- | --- | --- | --- |
| LSZ (0 μg/mL) + L-Glu (0 mM) vs. LSZ (0 μg/mL) + L-Glu (20 mM) | Yes | **** | <0.0001 |
| LSZ (0 μg/mL) + L-Glu (20 mM) vs. LSZ (10 μg/mL) + L-Glu (20 mM) | Yes | *** | 0.0009 |
| LSZ (0 μg/mL) vs. LSZ (10 μg/mL) | No | ns | 0.9405 |
| LSZ (10 μg/mL) vs. LSZ (10 μg/mL) + L-Glu (20 mM) | Yes | ** | 0.0062 |

**Figure 2B** Bcl-2 protein expression

| **One-way anova test** | **Significant?** | **Summary** | **Adjusted P Value** |
| --- | --- | --- | --- |
| LSZ (0 μg/mL) + L-Glu (0 mM) vs. LSZ (0 μg/mL) + L-Glu (20 mM) | No | ns | 0.1984 |
| LSZ (0 μg/mL) + L-Glu (20 mM) vs. LSZ (10 μg/mL) + L-Glu (20 mM) | Yes | *** | 0.0002 |
| LSZ (0 μg/mL) + L-Glu (20 mM) vs. LSZ (20 μg/mL) + L-Glu (20 mM) | Yes | ** | 0.0015 |

**Figure 2B** Bad protein expression

| **One-way anova test** | **Significant?** | **Summary** | **Adjusted P Value** |
| --- | --- | --- | --- |
| LSZ (0 μg/mL) + L-Glu (0 mM) vs. LSZ (0 μg/mL) + L-Glu (20 mM) | No | ns | 0.6742 |
| LSZ (0 μg/mL) + L-Glu (20 mM) vs. LSZ (10 μg/mL) + L-Glu (20 mM) | Yes | **** | <0.0001 |
| LSZ (0 μg/mL) + L-Glu (20 mM) vs. LSZ (20 μg/mL) + L-Glu (20 mM) | Yes | **** | <0.0001 |

**Figure 2B** p53 protein expression

| **One-way anova test** | **Significant?** | **Summary** | **Adjusted P Value** |
| --- | --- | --- | --- |
| LSZ (0 μg/mL) + L-Glu (0 mM) vs. LSZ (0 μg/mL) + L-Glu (20 mM) | Yes | *** | 0.001 |
| LSZ (0 μg/mL) + L-Glu (20 mM) vs. LSZ (10 μg/mL) + L-Glu (20 mM) | Yes | **** | <0.0001 |
| LSZ (0 μg/mL) + L-Glu (20 mM) vs. LSZ (20 μg/mL) + L-Glu (20 mM) | Yes | **** | <0.0001 |

**Figure 2C** Bax protein expression

| **One-way anova test** | **Significant?** | **Summary** | **Adjusted P Value** |
| --- | --- | --- | --- |
| LSZ (0 μg/mL) + L-Glu (0 mM) vs. LSZ (0 μg/mL) + L-Glu (20 mM) | Yes | * | 0.0152 |
| LSZ (0 μg/mL) + L-Glu (20 mM) vs. LSZ (10 μg/mL) + L-Glu (20 mM) | Yes | ** | 0.0026 |
| LSZ (0 μg/mL) + L-Glu (20 mM) vs. LSZ (20 μg/mL) + L-Glu (20 mM) | No | ns | 0.0874 |

**Figure 2C** NF-κB protein expression

| **One-way anova test** | **Significant?** | **Summary** | **Adjusted P Value** |
| --- | --- | --- | --- |
| LSZ (0 μg/mL) + L-Glu (0 mM) vs. LSZ (0 μg/mL) + L-Glu (20 mM) | Yes | *** | 0.0010 |
| LSZ (0 μg/mL) + L-Glu (20 mM) vs. LSZ (10 μg/mL) + L-Glu (20 mM) | Yes | ** | 0.0013 |
| LSZ (0 μg/mL) + L-Glu (20 mM) vs. LSZ (20 μg/mL) + L-Glu (20 mM) | Yes | ** | 0.0013 |

**Figure 2C** IL-1β protein expression

| **One-way anova test** | **Significant?** | **Summary** | **Adjusted P Value** |
| --- | --- | --- | --- |
| LSZ (0 μg/mL) + L-Glu (0 mM) vs. LSZ (0 μg/mL) + L-Glu (20 mM) | Yes | **** | <0.0001 |
| LSZ (0 μg/mL) + L-Glu (20 mM) vs. LSZ (10 μg/mL) + L-Glu (20 mM) | Yes | **** | <0.0001 |
| LSZ (0 μg/mL) + L-Glu (20 mM) vs. LSZ (20 μg/mL) + L-Glu (20 mM) | Yes | **** | <0.0001 |

**Figure 3C** Escape latency

| **One-way anova test** | **Significant?** | **Summary** | **Adjusted P Value** |
| --- | --- | --- | --- |
| WT vs. AC | Yes | **** | <0.0001 |
| AC vs. ALN | Yes | **** | <0.0001 |
| AC vs. ALH | Yes | **** | <0.0001 |

**Figure 3D** Platform crossings

| **One-way anova test** | **Significant?** | **Summary** | **Adjusted P Value** |
| --- | --- | --- | --- |
| WT vs. AC | Yes | *** | 0.0002 |
| AC vs. ALN | Yes | ** | 0.0068 |
| AC vs. ALH | No | ns | 0.5222 |

**Figure 3E** Times of passes through the target quadrant

| **One-way anova test** | **Significant?** | **Summary** | **Adjusted P Value** |
| --- | --- | --- | --- |
| WT vs. AC | Yes | *** | 0.0005 |
| AC vs. ALN | Yes | ** | 0.0016 |
| AC vs. ALH | Yes | * | 0.0129 |

**Figure 3G** New arm crossings

| **One-way anova test** | **Significant?** | **Summary** | **Adjusted P Value** |
| --- | --- | --- | --- |
| WT vs. AC | Yes | **** | <0.0001 |
| AC vs. ALN | Yes | ** | 0.0046 |
| AC vs. ALH | No | ns | 0.6329 |

**Figure 3H** Distance in new arm

| **One-way anova test** | **Significant?** | **Summary** | **Adjusted P Value** |
| --- | --- | --- | --- |
| WT vs. AC | Yes | *** | 0.0001 |
| AC vs. ALN | Yes | ** | 0.0091 |
| AC vs. ALH | No | ns | 0.0673 |

**Figure 3I** Time in new arm

| **One-way anova test** | **Significant?** | **Summary** | **Adjusted P Value** |
| --- | --- | --- | --- |
| WT vs. AC | Yes | **** | <0.0001 |
| AC vs. ALN | Yes | * | 0.0217 |
| AC vs. ALH | No | ns | 0.9006 |

**Figure 4A** TUNEL positive

| **One-way anova test** | **Significant?** | **Summary** | **Adjusted P Value** |
| --- | --- | --- | --- |
| WT vs. AC | Yes | **** | <0.0001 |
| AC vs. ALN | Yes | **** | <0.0001 |
| AC vs. ALH | Yes | **** | <0.0001 |

**Figure 4B** TUNEL positive

| **One-way anova test** | **Significant?** | **Summary** | **Adjusted P Value** |
| --- | --- | --- | --- |
| WT vs. AC | Yes | *** | 0.0003 |
| AC vs. ALN | Yes | **** | <0.0001 |
| AC vs. ALH | Yes | **** | <0.0001 |

**Figure 4C** Bad mRNA level

| **One-way anova test** | **Significant?** | **Summary** | **Adjusted P Value** |
| --- | --- | --- | --- |
| WT vs. AC | No | ns | 0.9834 |
| AC vs. ALN | Yes | *** | 0.0002 |
| AC vs. ALH | Yes | **** | <0.0001 |

**Figure 4C** Bax mRNA level

| **One-way anova test** | **Significant?** | **Summary** | **Adjusted P Value** |
| --- | --- | --- | --- |
| WT vs. AC | Yes | **** | <0.0001 |
| AC vs. ALN | Yes | ** | 0.0011 |
| AC vs. ALH | Yes | * | 0.0324 |

**Figure 4C** Bcl-2 mRNA level

| **One-way anova test** | **Significant?** | **Summary** | **Adjusted P Value** |
| --- | --- | --- | --- |
| WT vs. AC | No | ns | 0.8263 |
| AC vs. ALN | Yes | **** | <0.0001 |
| AC vs. ALH | Yes | **** | <0.0001 |

**Figure 4C** Bcl-xL mRNA level

| **One-way anova test** | **Significant?** | **Summary** | **Adjusted P Value** |
| --- | --- | --- | --- |
| WT vs. AC | Yes | **** | <0.0001 |
| AC vs. ALN | No | ns | 0.8055 |
| AC vs. ALH | Yes | **** | <0.0001 |

**Figure 4D** GSH-PX mRNA level

| **One-way anova test** | **Significant?** | **Summary** | **Adjusted P Value** |
| --- | --- | --- | --- |
| WT vs. AC | No | ns | 0.3750 |
| AC vs. ALN | Yes | **** | <0.0001 |
| AC vs. ALH | Yes | **** | <0.0001 |

**Figure 4D** SOD1 mRNA level

| **One-way anova test** | **Significant?** | **Summary** | **Adjusted P Value** |
| --- | --- | --- | --- |
| WT vs. AC | No | ns | 0.6947 |
| AC vs. ALN | No | ns | 0.0551 |
| AC vs. ALH | Yes | **** | <0.0001 |

**Figure 4D** SOD2 mRNA level

| **One-way anova test** | **Significant?** | **Summary** | **Adjusted P Value** |
| --- | --- | --- | --- |
| WT vs. AC | No | ns | 0.6974 |
| AC vs. ALN | Yes | * | 0.0194 |
| AC vs. ALH | Yes | **** | <0.0001 |

**Figure 5A** PSEN1 MFI/cell number

| **One-way anova test** | **Significant?** | **Summary** | **Adjusted P Value** |
| --- | --- | --- | --- |
| WT vs. AC | Yes | **** | <0.0001 |
| AC vs. ALN | Yes | **** | <0.0001 |
| AC vs. ALH | Yes | **** | <0.0001 |

**Figure 5C** Thioflavin S MFI

| **One-way anova test** | **Significant?** | **Summary** | **Adjusted P Value** |
| --- | --- | --- | --- |
| WT vs. AC | Yes | **** | <0.0001 |
| AC vs. ALN | Yes | **** | <0.0001 |
| AC vs. ALH | Yes | **** | <0.0001 |

**Figure 5D** BACE1 mRNA level

| **One-way anova test** | **Significant?** | **Summary** | **Adjusted P Value** |
| --- | --- | --- | --- |
| WT vs. AC | Yes | **** | <0.0001 |
| AC vs. ALN | Yes | **** | <0.0001 |
| AC vs. ALH | Yes | **** | <0.0001 |

**Figure 5D** APH1a mRNA level

| **One-way anova test** | **Significant?** | **Summary** | **Adjusted P Value** |
| --- | --- | --- | --- |
| WT vs. AC | Yes | *** | 0.0003 |
| AC vs. ALN | Yes | *** | 0.0001 |
| AC vs. ALH | Yes | *** | 0.0001 |

**Figure 5D** PEN2 mRNA level

| **One-way anova test** | **Significant?** | **Summary** | **Adjusted P Value** |
| --- | --- | --- | --- |
| WT vs. AC | Yes | **** | <0.0001 |
| AC vs. ALN | Yes | **** | <0.0001 |
| AC vs. ALH | Yes | **** | <0.0001 |

**Figure 6A** NF-κB MFI/cell number

| **One-way anova test** | **Significant?** | **Summary** | **Adjusted P Value** |
| --- | --- | --- | --- |
| WT vs. AC | Yes | **** | <0.0001 |
| AC vs. ALN | Yes | **** | <0.0001 |
| AC vs. ALH | Yes | **** | <0.0001 |

**Figure 6B** IL-1β MFI/cell number

| **One-way anova test** | **Significant?** | **Summary** | **Adjusted P Value** |
| --- | --- | --- | --- |
| WT vs. AC | Yes | **** | <0.0001 |
| AC vs. ALN | Yes | **** | <0.0001 |
| AC vs. ALH | Yes | **** | <0.0001 |

**Figure 6C** IL-10 mRNA level

| **One-way anova test** | **Significant?** | **Summary** | **Adjusted P Value** |
| --- | --- | --- | --- |
| WT vs. AC | Yes | ** | 0.0065 |
| AC vs. ALN | No | ns | 0.9853 |
| AC vs. ALH | Yes | *** | 0.0001 |

**Figure 6C** IL-1β mRNA level

| **One-way anova test** | **Significant?** | **Summary** | **Adjusted P Value** |
| --- | --- | --- | --- |
| WT vs. AC | Yes | *** | 0.0004 |
| AC vs. ALN | Yes | **** | <0.0001 |
| AC vs. ALH | Yes | **** | <0.0001 |

**Figure 6C** IL-6 mRNA level

| **One-way anova test** | **Significant?** | **Summary** | **Adjusted P Value** |
| --- | --- | --- | --- |
| WT vs. AC | Yes | * | 0.0192 |
| AC vs. ALN | Yes | **** | <0.0001 |
| AC vs. ALH | Yes | **** | <0.0001 |

**Figure 6C** TNF mRNA level

| **One-way anova test** | **Significant?** | **Summary** | **Adjusted P Value** |
| --- | --- | --- | --- |
| WT vs. AC | Yes | * | 0.0325 |
| AC vs. ALN | Yes | * | 0.0459 |
| AC vs. ALH | Yes | * | 0.0323 |

**Figure 7A** Iba-1 MFI/cell number

| **One-way anova test** | **Significant?** | **Summary** | **Adjusted P Value** |
| --- | --- | --- | --- |
| WT vs. AC | Yes | **** | <0.0001 |
| AC vs. ALN | Yes | **** | <0.0001 |
| AC vs. ALH | Yes | **** | <0.0001 |

**Figure 7B** p-Tau MFI/cell number

| **One-way anova test** | **Significant?** | **Summary** | **Adjusted P Value** |
| --- | --- | --- | --- |
| WT vs. AC | Yes | **** | <0.0001 |
| AC vs. ALN | Yes | **** | <0.0001 |
| AC vs. ALH | Yes | **** | <0.0001 |

**Figure 7C** p-Tau/total-Tau

| **One-way anova test** | **Significant?** | **Summary** | **Adjusted P Value** |
| --- | --- | --- | --- |
| WT vs. AC | Yes | **** | <0.0001 |
| AC vs. ALN | Yes | **** | <0.0001 |
| AC vs. ALH | Yes | **** | <0.0001 |

**Supplementary Figure 1A** FAS mRNA level

| **Two-way anova test** | | **Significant?** | **Summary** | **Adjusted P Value** |
| --- | --- | --- | --- | --- |
| siCtrl | LSZ (0 μg/mL) + L-Glu (0 mM)  vs. LSZ (0 μg/mL) + L-Glu (20 mM) | Yes | **** | <0.0001 |
|  | LSZ (0 μg/mL) + L-Glu (20 mM)  vs. LSZ (10 μg/mL) + L-Glu (20 mM) | Yes | **** | <0.0001 |
|  | LSZ (0 μg/mL) + L-Glu (20 mM) vs. LSZ (20 μg/mL) + L-Glu (20 mM) | Yes | **** | <0.0001 |
| siFAS | LSZ (0 μg/mL) + L-Glu (0 mM)  vs. LSZ (0 μg/mL) + L-Glu (20 mM) | No | ns | 0.0733 |
|  | LSZ (0 μg/mL) + L-Glu (20 mM)  vs. LSZ (10 μg/mL) + L-Glu (20 mM) | No | ns | 0.9959 |
|  | LSZ (0 μg/mL) + L-Glu (20 mM) vs. LSZ (20 μg/mL) + L-Glu (20 mM) | No | ns | 0.1057 |
| siCtrl- LSZ (0 μg/mL) + L-Glu (0 mM)  vs. siFAS-LSZ (0 μg/mL) + L-Glu (0 mM) | | Yes | **** | <0.0001 |

**Supplementary Figure 1A** Bcl-xL mRNA level

| **Two-way anova test** | | **Significant?** | **Summary** | **Adjusted P Value** |
| --- | --- | --- | --- | --- |
| siCtrl | LSZ (0 μg/mL) + L-Glu (0 mM)  vs. LSZ (0 μg/mL) + L-Glu (20 mM) | Yes | **** | <0.0001 |
|  | LSZ (0 μg/mL) + L-Glu (20 mM)  vs. LSZ (10 μg/mL) + L-Glu (20 mM) | Yes | **** | <0.0001 |
|  | LSZ (0 μg/mL) + L-Glu (20 mM) vs. LSZ (20 μg/mL) + L-Glu (20 mM) | Yes | **** | <0.0001 |
| siFAS | LSZ (0 μg/mL) + L-Glu (0 mM)  vs. LSZ (0 μg/mL) + L-Glu (20 mM) | Yes | **** | <0.0001 |
|  | LSZ (0 μg/mL) + L-Glu (20 mM)  vs. LSZ (10 μg/mL) + L-Glu (20 mM) | No | ns | 0.9956 |
|  | LSZ (0 μg/mL) + L-Glu (20 mM) vs. LSZ (20 μg/mL) + L-Glu (20 mM) | No | ns | 0.9995 |
| siCtrl- LSZ (0 μg/mL) + L-Glu (0 mM)  vs. siFAS-LSZ (0 μg/mL) + L-Glu (0 mM) | | Yes | ** | 0.0019 |

**Supplementary Figure 1A** Bad mRNA level

| **Two-way anova test** | | **Significant?** | **Summary** | **Adjusted P Value** |
| --- | --- | --- | --- | --- |
| siCtrl | LSZ (0 μg/mL) + L-Glu (0 mM)  vs. LSZ (0 μg/mL) + L-Glu (20 mM) | Yes | **** | <0.0001 |
|  | LSZ (0 μg/mL) + L-Glu (20 mM)  vs. LSZ (10 μg/mL) + L-Glu (20 mM) | Yes | **** | <0.0001 |
|  | LSZ (0 μg/mL) + L-Glu (20 mM) vs. LSZ (20 μg/mL) + L-Glu (20 mM) | Yes | **** | <0.0001 |
| siFAS | LSZ (0 μg/mL) + L-Glu (0 mM)  vs. LSZ (0 μg/mL) + L-Glu (20 mM) | Yes | ** | 0.0021 |
|  | LSZ (0 μg/mL) + L-Glu (20 mM)  vs. LSZ (10 μg/mL) + L-Glu (20 mM) | No | ns | 0.9994 |
|  | LSZ (0 μg/mL) + L-Glu (20 mM) vs. LSZ (20 μg/mL) + L-Glu (20 mM) | No | ns | 0.2227 |
| siCtrl- LSZ (0 μg/mL) + L-Glu (0 mM)  vs. siFAS-LSZ (0 μg/mL) + L-Glu (0 mM) | | Yes | **** | <0.0001 |

**Supplementary Figure 1A** Bax mRNA level

| **Two-way anova test** | | **Significant?** | **Summary** | **Adjusted P Value** |
| --- | --- | --- | --- | --- |
| siCtrl | LSZ (0 μg/mL) + L-Glu (0 mM)  vs. LSZ (0 μg/mL) + L-Glu (20 mM) | Yes | **** | <0.0001 |
|  | LSZ (0 μg/mL) + L-Glu (20 mM)  vs. LSZ (10 μg/mL) + L-Glu (20 mM) | Yes | **** | <0.0001 |
|  | LSZ (0 μg/mL) + L-Glu (20 mM) vs. LSZ (20 μg/mL) + L-Glu (20 mM) | Yes | **** | <0.0001 |
| siFAS | LSZ (0 μg/mL) + L-Glu (0 mM)  vs. LSZ (0 μg/mL) + L-Glu (20 mM) | No | ns | 0.4345 |
|  | LSZ (0 μg/mL) + L-Glu (20 mM)  vs. LSZ (10 μg/mL) + L-Glu (20 mM) | No | ns | 0.4596 |
|  | LSZ (0 μg/mL) + L-Glu (20 mM) vs. LSZ (20 μg/mL) + L-Glu (20 mM) | No | ns | 0.2604 |
| siCtrl- LSZ (0 μg/mL) + L-Glu (0 mM)  vs. siFAS-LSZ (0 μg/mL) + L-Glu (0 mM) | | Yes | **** | <0.0001 |

**Supplementary Figure 1C** FAS protein expression

| **Two-way anova test** | | **Significant?** | **Summary** | **Adjusted P Value** |
| --- | --- | --- | --- | --- |
| siCtrl | LSZ (0 μg/mL) + L-Glu (0 mM)  vs. LSZ (0 μg/mL) + L-Glu (20 mM) | Yes | **** | <0.0001 |
|  | LSZ (0 μg/mL) + L-Glu (20 mM)  vs. LSZ (10 μg/mL) + L-Glu (20 mM) | Yes | **** | <0.0001 |
|  | LSZ (0 μg/mL) + L-Glu (20 mM) vs. LSZ (20 μg/mL) + L-Glu (20 mM) | Yes | **** | <0.0001 |
| siFAS | LSZ (0 μg/mL) + L-Glu (0 mM)  vs. LSZ (0 μg/mL) + L-Glu (20 mM) | No | ns | 0.5481 |
|  | LSZ (0 μg/mL) + L-Glu (20 mM)  vs. LSZ (10 μg/mL) + L-Glu (20 mM) | No | ns | 0.6431 |
|  | LSZ (0 μg/mL) + L-Glu (20 mM) vs. LSZ (20 μg/mL) + L-Glu (20 mM) | No | ns | 0.9999 |
| siCtrl- LSZ (0 μg/mL) + L-Glu (0 mM)  vs. siFAS-LSZ (0 μg/mL) + L-Glu (0 mM) | | Yes | **** | <0.0001 |

**Supplementary Figure 1C** Bax protein expression

| **Two-way anova test** | | **Significant?** | **Summary** | **Adjusted P Value** |
| --- | --- | --- | --- | --- |
| siCtrl | LSZ (0 μg/mL) + L-Glu (0 mM)  vs. LSZ (0 μg/mL) + L-Glu (20 mM) | Yes | ** | 0.0049 |
|  | LSZ (0 μg/mL) + L-Glu (20 mM)  vs. LSZ (10 μg/mL) + L-Glu (20 mM) | Yes | **** | <0.0001 |
|  | LSZ (0 μg/mL) + L-Glu (20 mM) vs. LSZ (20 μg/mL) + L-Glu (20 mM) | Yes | **** | <0.0001 |
| siFAS | LSZ (0 μg/mL) + L-Glu (0 mM)  vs. LSZ (0 μg/mL) + L-Glu (20 mM) | No | ns | 0.9870 |
|  | LSZ (0 μg/mL) + L-Glu (20 mM)  vs. LSZ (10 μg/mL) + L-Glu (20 mM) | No | ns | 0.9989 |
|  | LSZ (0 μg/mL) + L-Glu (20 mM) vs. LSZ (20 μg/mL) + L-Glu (20 mM) | No | ns | 0.4042 |
| siCtrl- LSZ (0 μg/mL) + L-Glu (0 mM)  vs. siFAS-LSZ (0 μg/mL) + L-Glu (0 mM) | | Yes | **** | <0.0001 |

**Supplementary Figure 1C** Bcl-2 protein expression

| **Two-way anova test** | | **Significant?** | **Summary** | **Adjusted P Value** |
| --- | --- | --- | --- | --- |
| siCtrl | LSZ (0 μg/mL) + L-Glu (0 mM)  vs. LSZ (0 μg/mL) + L-Glu (20 mM) | Yes | **** | <0.0001 |
|  | LSZ (0 μg/mL) + L-Glu (20 mM)  vs. LSZ (10 μg/mL) + L-Glu (20 mM) | Yes | **** | <0.0001 |
|  | LSZ (0 μg/mL) + L-Glu (20 mM) vs. LSZ (20 μg/mL) + L-Glu (20 mM) | Yes | **** | <0.0001 |
| siFAS | LSZ (0 μg/mL) + L-Glu (0 mM)  vs. LSZ (0 μg/mL) + L-Glu (20 mM) | No | ns | 0.4074 |
|  | LSZ (0 μg/mL) + L-Glu (20 mM)  vs. LSZ (10 μg/mL) + L-Glu (20 mM) | No | ns | 0.8768 |
|  | LSZ (0 μg/mL) + L-Glu (20 mM) vs. LSZ (20 μg/mL) + L-Glu (20 mM) | No | ns | 0.9998 |
| siCtrl- LSZ (0 μg/mL) + L-Glu (0 mM)  vs. siFAS-LSZ (0 μg/mL) + L-Glu (0 mM) | | Yes | **** | <0.0001 |

# Supplementary Figures and Tables

## Supplementary Figures


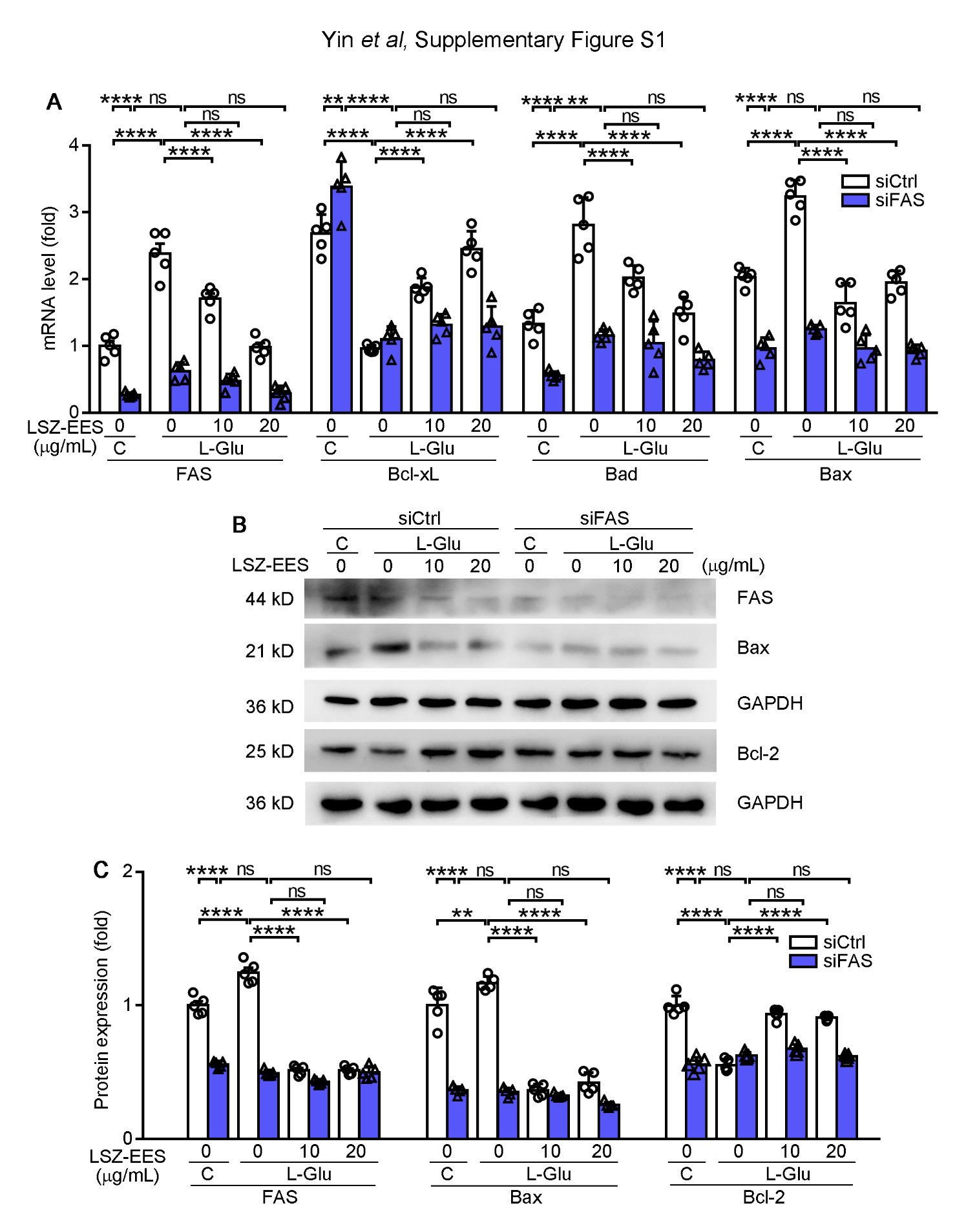


**Supplementary Figure S1. LSZ antagonizes L-Glu-induced neuron apoptosis partly through FAS/Bcl-2/p53 pathway**

After FAS siRNA (siFAS) or negative control siRNA (siCtrl) transfection, HT-22 cells were pre-treated with LSZ-EES (10 μg/mL) for 3 h followed by co-treatment with 20 mM L-Glu for another 18 h; expression of FAS, Bcl-xL, Bad and Bax mRNA was determined by qRT-PCR (A); expression of FAS, Bax and Bcl-2 protein was detected by Western blot (B) with quantitative analysis of band density and normalization to GAPDH (C). *p < 0.05, **p < 0.01, ***p < 0.001, ****p < 0.0001. ns, not significant (n = 5).


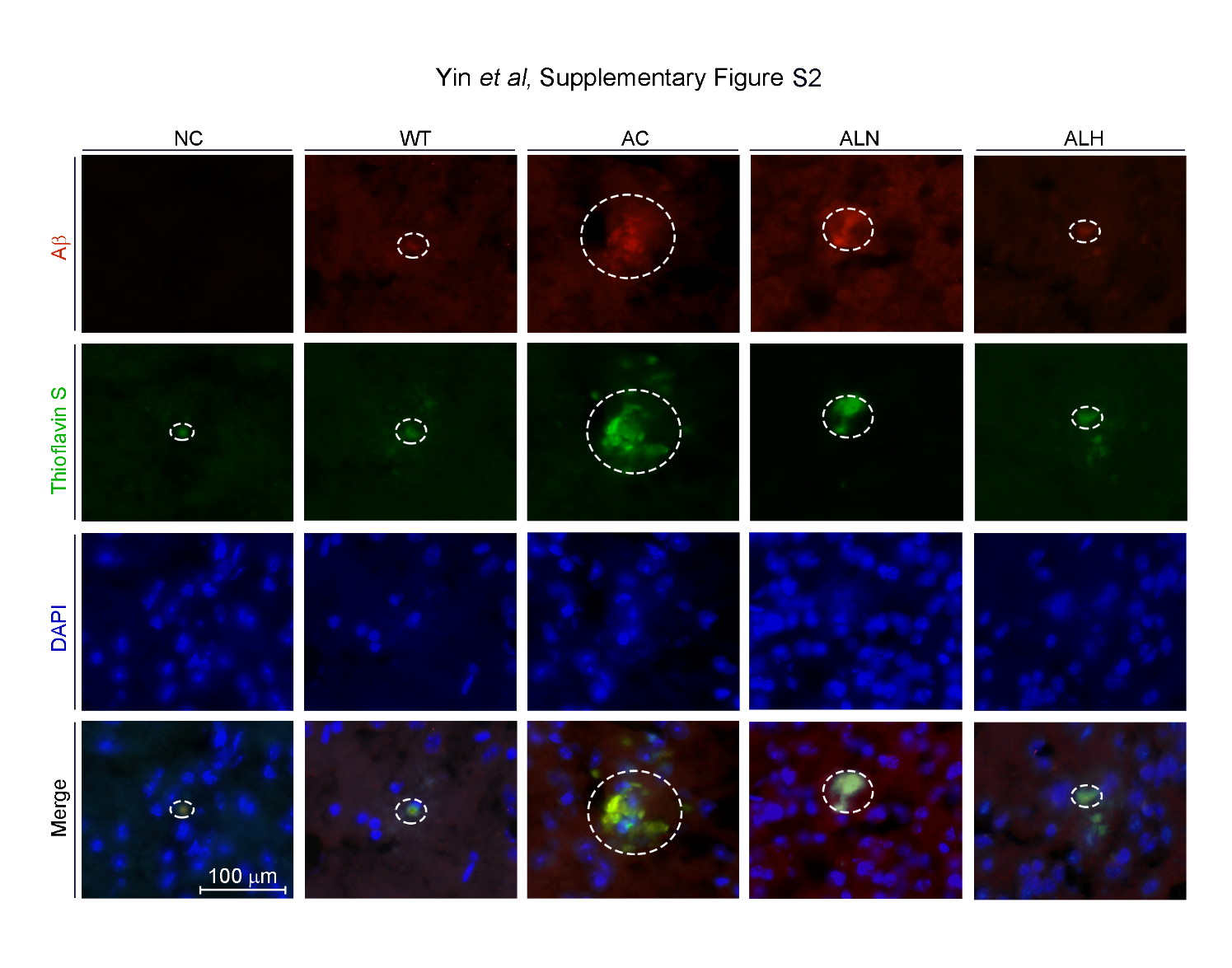


**Supplementary Figure 2.** **LSZ reduces the accumulation of amyloid plaques in the brains of APP/PS1 mice.**

Amyloid plaque deposits in mouse brain was determined by co-staining with Aβ antibody (red) and thioflavin S (green). The nuclear was stained with DAPI (blue). NC: negative control; (scale bar 100 μm).

## Uncropped Images of Western Blot


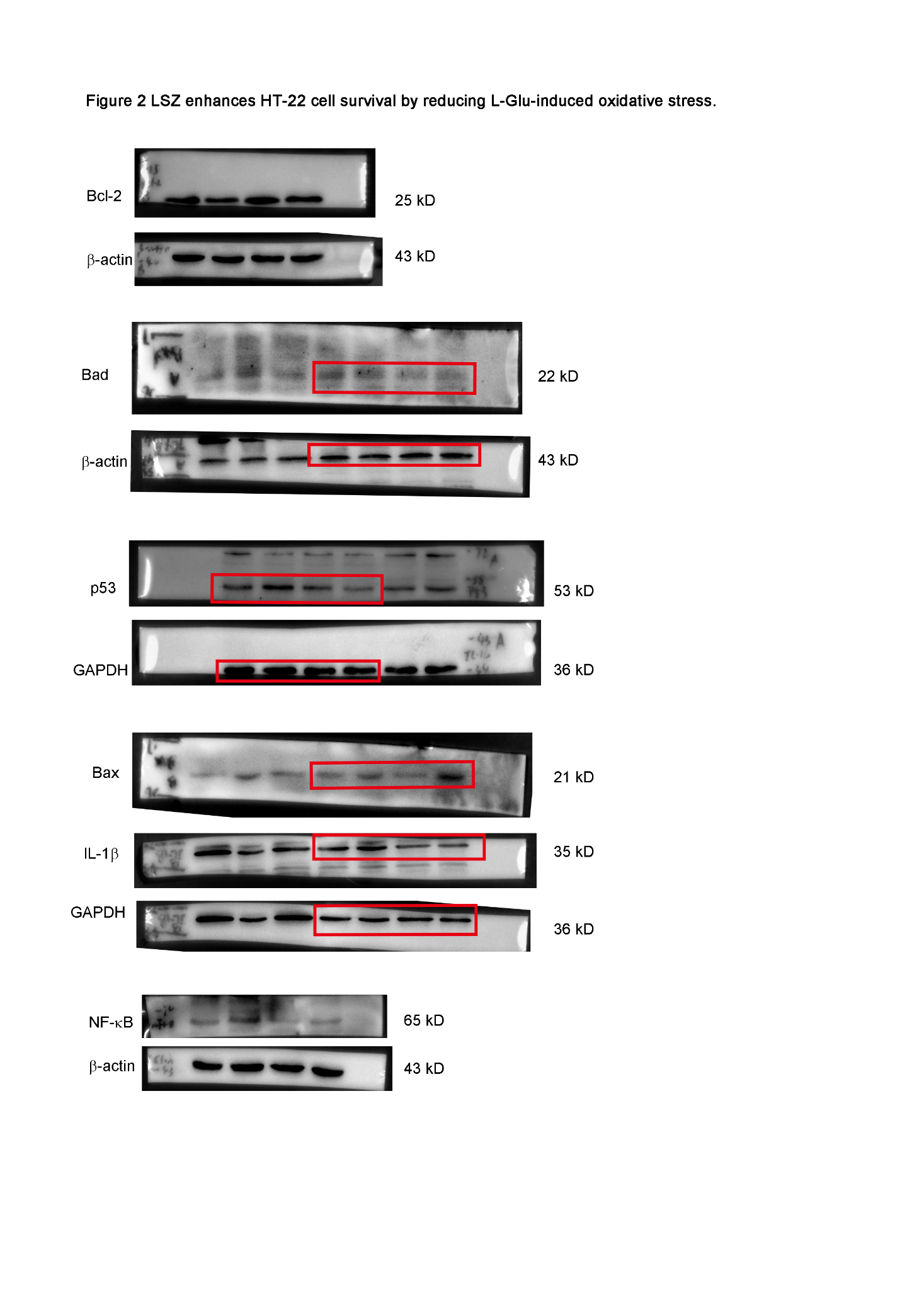


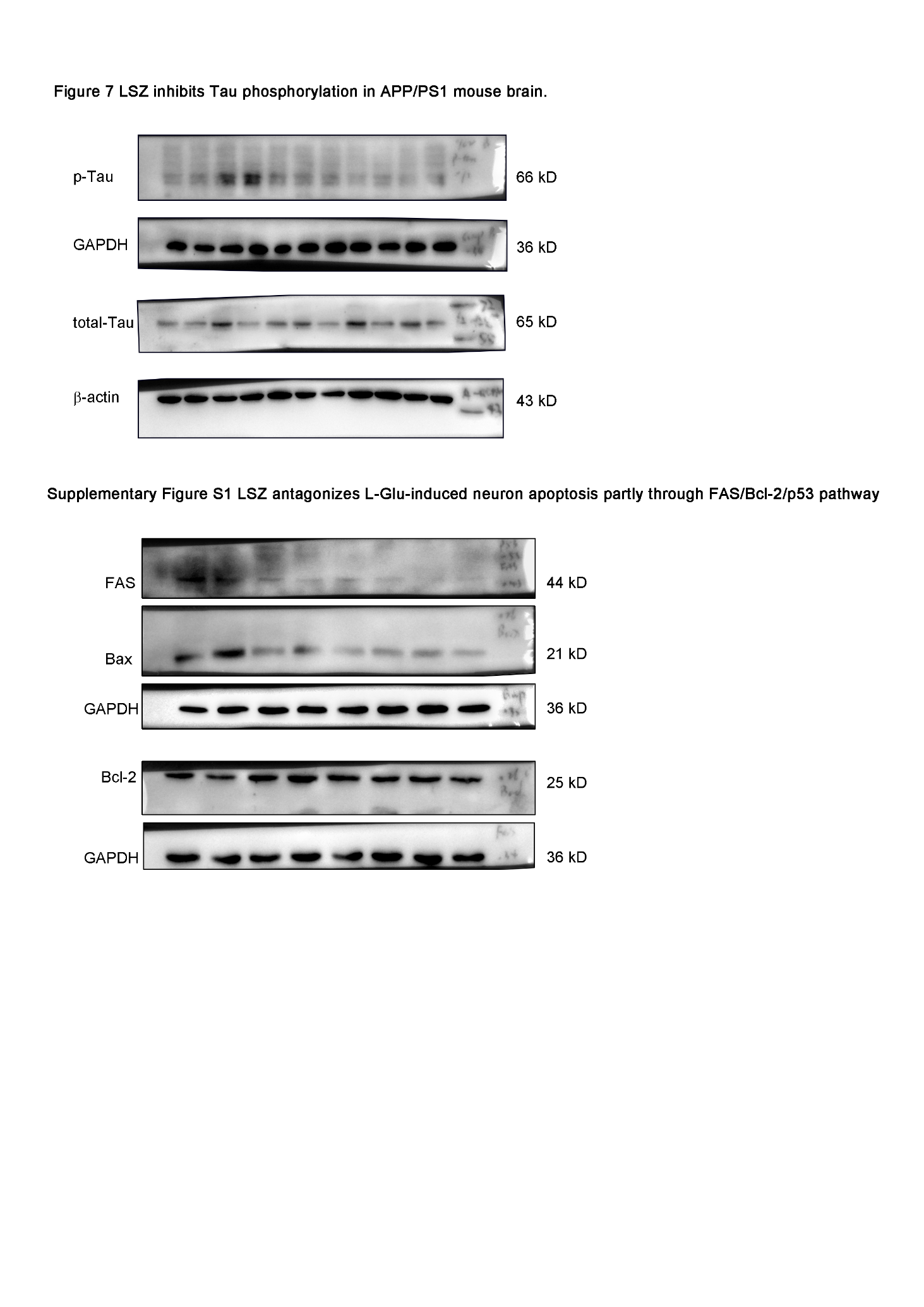

Supplement: Supplementary file 1 [file Data_Sheet_1.DOCX]
